# BG2: Bayesian variable selection in generalized linear mixed models with nonlocal priors for non-Gaussian GWAS data

**DOI:** 10.1186/s12859-023-05468-w

**Published:** 2023-09-15

**Authors:** Shuangshuang Xu, Jacob Williams, Marco A. R. Ferreira

**Affiliations:** Department of Statistics, Virginia Tech, Blacksburg, VA 24061 USA

**Keywords:** Bayesian statistics, GLMM, GWAS, Nonlocal prior, Variable selection

## Abstract

**Background:**

Genome-wide association studies (GWASes) aim to identify single nucleotide polymorphisms (SNPs) associated with a given phenotype. A common approach for the analysis of GWAS is single marker analysis (SMA) based on linear mixed models (LMMs). However, LMM-based SMA usually yields a large number of false discoveries and cannot be directly applied to non-Gaussian phenotypes such as count data.

**Results:**

We present a novel Bayesian method to find SNPs associated with non-Gaussian phenotypes. To that end, we use generalized linear mixed models (GLMMs) and, thus, call our method Bayesian GLMMs for GWAS (BG2). To deal with the high dimensionality of GWAS analysis, we propose novel nonlocal priors specifically tailored for GLMMs. In addition, we develop related fast approximate Bayesian computations. BG2 uses a two-step procedure: first, BG2 screens for candidate SNPs; second, BG2 performs model selection that considers all screened candidate SNPs as possible regressors. A simulation study shows favorable performance of BG2 when compared to GLMM-based SMA. We illustrate the usefulness and flexibility of BG2 with three case studies on cocaine dependence (binary data), alcohol consumption (count data), and number of root-like structures in a model plant (count data).

**Supplementary Information:**

The online version contains supplementary material available at 10.1186/s12859-023-05468-w.

## Introduction

Genome-wide association studies (GWAS) have uncovered many single nucleotide polymorphisms (SNP) associated to important phenotypes such as plant productivity [1], plant response to salt stress [2], and human diseases [3]. To take into account the correlation among GWAS observations, the most widely used methods for the analysis of GWAS continuous Gaussian data are single marker analysis (SMA) methods based on linear mixed models (LMMs) [4–6]. Recently, SMA based on logistic regression with random effects has been proposed for the analysis of GWAS binary data [7]. However, to the best of our knowledge, there are no published methods for the analysis of other types of *correlated GWAS non-Gaussian* data such as count data. One of our contributions is to propose the use of generalized linear mixed models for the analysis of GWAS non-Gaussian data. To that end, we use generalized linear mixed models (GLMMs) and, thus, call our method Bayesian GLMMs for GWAS (BG2).

BG2 has two steps: a screening step and a model selection step. The screening step, similarly to SMA methods, fits *p* GLMMs where each model has just one SNP, and uses Bayesian FDR control [8, 9] to provide a set of candidate SNPs. After that, the model selection step performs a model search through the space of GLMMs that may include any number of screened candidate SNPs as possible regressors. BG2 implements both steps using a pseudo-likelihood approach. We note that a similar pseudo-likelihood approach can be used to implement SMA methods for non-Gaussian GWAS data, and a particular case of such an approach has been proposed for GWAS binary data [7]. However, simulation studies presented in Sect. 4 show that, when compared to such SMA methods for non-Gaussian data, BG2 leads to much lower FDR.

The GLMMs for GWAS data considered by BG2 may have two types of random effects: kinship randoms effects and overdispersion random effects. The kinship random effects account for correlation among GWAS observations due to population stratification and hidden relatedness. Similarly to existing literature for Gaussian GWAS data, we assume that the vector of kinship random effects follows a multivariate Gaussian distribution with a mean vector of zeros and a covariance matrix that is the product of a one-dimensional unknown variance parameter and a known positive semi-definite kinship matrix [10, 11]. The overdispersion random effects allow for extra variability not accounted for by the model for observations; for example, when assuming a conditional Poisson model for the observations, the overdispersion random effects account for extra-Poisson variability.

Both screening and model selection steps in BG2 are based on nonlocal priors. To the best of our knowledge, this is the first time that nonlocal priors are proposed for regression coefficients in GLMMs. Previous literature in Bayesian model selection for GLMMs has assigned for regression coefficients local priors [12]. While local priors have positive density at null parameter values, nonlocal priors have density equal to zero at null parameter values. Nonlocal priors were first proposed by [13, 14] for Gaussian linear models. Nonlocal priors have been successfully developed for many different problems such as model selection in Gaussian directed acyclic graphical models [15], classification with Bayesian probit models [16], variable selection in logistic models [17], Bayesian wavelet analysis [18], and variable selection in generalized linear models [19]. In particular, [20, 21] have proposed methods based on nonlocal priors for variable selection in linear mixed models applied to GWAS data. However, because LMMs applied to binary or count data may lead to meaningless negative predictions and statistically inefficient estimation, LMMs should not be applied to non-Gaussian data such as count and binary data, which are the types of data considered by BG2. Nonlocal priors lead to faster accumulation of evidence in favor of a true null hypothesis [13], and have been shown to be advantageous for high-dimensional problems [14, 16, 22]. Therefore, BG2 uses nonlocal priors for SNP search in GWAS analysis.

Due to the large number of GLMMs that need to be fitted, BG2 relies on two approximations to speed up computations: a pseudo-likelihood approximation; and a Population Parameters Previously Determined (P3D) approximation that may be seen as an empirical Bayes approach. For GLMMs, the integrated likelihood function obtained by integrating out the random effects is not available in closed form. Repeated numerical integration of the random effects for each GLMM fitted for a GWAS analysis is computationally too expensive. Thus, BG2 uses a pseudo-likelihood approach [23] to facilitate integrating out the random effects. Such pseudo-likelihood approach leads to a Gaussian approximation for adjusted observations that allows analytically integrating out the random effects. In addition, to avoid the computation of matrix inverses for each SNP and, thus, to further speed up computations, we propose a P3D approximation for GLMMs. A P3D approximation was first proposed by [24] for Gaussian linear mixed models (LMMs) and a variation of this approximation is used in the celebrated and widely used method EMMAX for the analysis of GWAS Gaussian data [6]. With our P3D approach, BG2 needs to compute a spectral decomposition only once for each screening step and only once for each model selection step.

In our P3D approach, for each BG2 step (screening and model selection) we fit a baseline GLMM to obtain adjusted observations and estimates of the variance parameters. We then keep the adjusted observations and the variance parameters fixed at the values computed with the baseline GLMM when fitting all other models in that BG2 step. In our P3D approach, the baseline model is different for the screening step and for the model selection step. For the screening step, the baseline model is a GLMM without any SNPs. For the model selection step, the baseline model is a GLMM with all candidate SNPs obtained from the screening step. This choice of baseline GLMM for the model selection step is based on [25], who have suggested for GLMMs the use of adjusted observations based on the full model – the model with all the regressors – when computing BICs for all possible models. Therefore, BG2 with our P3D approximation does not need to compute a spectral decomposition for each SNP. As a result, when compared to a usual pseudo-likelihood approach to GLMMs, our P3D approximation greatly reduces the computational time and allows the analysis of non-Gaussian GWAS data within a reasonable time frame.

To be technical, in this work we use a hierarchical model and an empirical Bayes approach to estimate the hyperparameters of the prior distribution of the regression coefficients of GLMMs. We then combine this prior distribution with the data through Bayes Theorem to compute the posterior probability of the competing GLMMs. The Bayesian model selection procedure that we propose in this work is similar to that of [26], except that in our current work we are dealing with the problem of Bayesian ultra-high dimensional variable selection (*p* two orders of magnitude larger than *n*) in GLMMs applied to GWAS analysis. To the best of our knowledge, currently there are no published methods for Bayesian ultra-high dimensional variable selection in GLMMs.

The remainder of this paper is organized as follows. Section 2 describes the GLMMs that we consider for non-Gaussian GWAS data. Section 3 describes our BG2 method for the identification of causal SNPs. Section 4 presents the results of two simulation studies for binary data and for count data. Section 5 illustrates our method with applications to three case studies: human cocaine dependence, alcohol consumption, and the number of root-like structures in the plant *A. Thaliana*. Section 6 concludes with a discussion and future directions.

## GLMMs for GWAS

Consider observations $y_1, \dots , y_n$ that, given random effects, are conditionally independent and have a distribution from the exponential family of distributions. This flexible family of distributions includes the Bernoulli, binomial, Poisson, and gamma distributions. Thus, this family may be used to model observed GWAS phenotypes such as an indicator of disease presence/absence, number of lateral roots in plants, or survival time. Then, the density function of $y_i$ is

1 $$\begin{aligned} f(y_i|\eta _i) = \exp [{ T(y_i)} \eta _i-B(\eta _i)+C(y_i)], \end{aligned}$$

for $i=1,\dots ,n$, where $T(y_i)$ is the sufficient statistics for $y_i$, *B*(.) and *C*(.) are known functions. Further, each observation $y_i$ has mean $\mu _i=B'(\eta _i)$ and variance $v_i = B''(\eta _i)$. Let $X_s$ be a matrix of SNPs and $\pmb {\beta }_s$ be the corresponding vector of regression coefficients. In addition, let $X_c$ be a matrix that contains a column of ones for the intercept and other columns for control covariates (e.g., age, sex, and environmental factors) and $\pmb {\beta }_c$ be the corresponding vector of regression coefficients. Thus, $\pmb {\beta }_s$ and $\pmb {\beta }_c$ are fixed effects. Further, let $\pmb {\alpha }_1$ be a vector of random effects that accounts for kinship correlation. Specifically, $\pmb {\alpha }_1$ has a multivariate normal distribution with mean vector $\pmb {0}$ and covariance matrix $\kappa _1 \Sigma$, where $\kappa _1$ is an unknown scalar and $\Sigma$ is a kinship matrix. Furthermore, let $\pmb {\alpha }_2$ be a vector of overdispersion random effects following $N(\pmb {0}, \kappa _2 I)$. Let $\pmb {y}=(y_1, \dots , y_n)$ be the vector of observed phenotypes. Then, the conditional expectation $E(\pmb {y}|\pmb {\alpha }_1,\pmb {\alpha }_2)$ is linked to the linear predictor $X_s\pmb {\beta }_s+X_c\pmb {\beta }_c+\pmb {\alpha }_1+\pmb {\alpha }_2$ by the link function *g*:

2 $$\begin{aligned} g(E(\pmb {y}|\pmb {\alpha }_1,\pmb {\alpha }_2)) = X_s\pmb {\beta }_s+X_c\pmb {\beta }_c+\pmb {\alpha }_1+\pmb {\alpha }_2. \end{aligned}$$

The class of GLMMs given by Eqs. (1) and (2) can be expanded to deal with other cases. For example, to account for the experimental design used for data collection, we may add another random effect $\pmb {\alpha }_3$ following a multivariate normal distribution with mean vector $\pmb {0}$ and covariance matrix $\kappa _3 \Sigma _3$, where $\kappa _3$ is a unknown parameter and $\Sigma _3$ is a symmetric positive semi-definite matrix that describes the dependence structure among the observations due to the experimental design. Because of the P3D approach, BG2 can include additional random effects and still use the spectral decomposition approach to speed up computations.

## BG2: Bayesian SNP selection in GLMMs for GWAS

Our method BG2 consists of two steps: screening and model selection. The BG2 screening step uses a novel Bayesian single marker analysis for non-Gaussian data and applies Bayesian false discovery rate control to yield a set of candidate SNPs. After that, the BG2 model selection step performs a search through the model space of all GLMMs that may include any number of SNPs from the set of candidate SNPs. In both steps, BG2 uses a pseudo-likelihood approach to fit models. In what follows, Sect. 3.1 presents the pseudo-likelihood approach, Sect. 3.2 introduces the BG2 screening step, and Sect. 3.3 presents the BG2 model selection step.

### Pseudo-likelihood model fitting

In both the screening and the model selection steps, BG2 uses a pseudo-likelihood approach. In this subsection, we provide a summary description of the pseudo-likelihood approach. In addition, Additional file 1: Section S1 provides a detailed presentation of the pseudo-likelihood approach. This is an iterative approach that writes the model for the observations as $\pmb {y} = \pmb {\mu } + \pmb {\epsilon }$, where $\pmb {\epsilon }$ is a vector of errors and $V=Var(\pmb {\epsilon })=Var(\pmb {y}) {= diag(v_1, \ldots , v_n)}$ is a diagonal matrix. Note that for distributions in the exponential family, the variance $v_i$ depends on the linear predictor $\eta _i$ and, thus, gets updated in each iteration of the pseudo-likelihood algorithm. More details can be found in Additional file 1: Section S1. In addition, the pseudo-likelihood approach expands $\pmb {\mu }=E(\pmb {y}|\pmb {\beta }_s,\pmb {\beta }_c, \pmb {\alpha }_1,\pmb {\alpha }_2)$ in a first-order Taylor expansion around current estimates of $\pmb {\beta }_s$, $\pmb {\beta }_c$, $\pmb {\alpha }_1$, $\pmb {\alpha }_2$, $\kappa _1$, and $\kappa _2$. The resulting equation is rearranged such that the left-hand side depends only on known quantities (observations, current estimates of parameters, regression matrices). Then, this equation is pre-multiplied by $V^{-1}$. Let ${\hat{V}}$ be the current estimate for *V*. The left-hand side of the resulting equation, known as the vector of adjusted observations, is $\pmb {y}^\star = {\widehat{V}}^{-1}(\pmb {y}-\widehat{\pmb {\mu }})+X_s\widehat{\pmb {\beta }}_s+ X_c\widehat{\pmb {\beta }}_c+ \widehat{\pmb {\alpha }}_1+\widehat{\pmb {\alpha }}_2$. Equating $\pmb {y}^\star$ to the right-hand side of the resulting equation yields

3 $$\begin{aligned} \pmb {y}^\star = X_s \pmb {\beta }_s + X_c \pmb {\beta }_c + \pmb {\alpha }_1 + \pmb {\alpha }_2 + {\widehat{V}}^{-1}\pmb {\epsilon }. \end{aligned}$$

Then, the pseudo-likelihood approach approximates the GLMM by an LMM given by Eq. (3) with vectors of random effects $\pmb {\alpha }_1 \sim N(\pmb {0}, \kappa _1\Sigma )$ and $\pmb {\alpha }_2 \sim N(\pmb {0}, \kappa _2 I).$ Based on this LMM, new estimates are computed for $\pmb {\beta }_s$, $\pmb {\beta }_c$, $\pmb {\alpha }_1$, $\pmb {\alpha }_2$, $\kappa _1$, $\kappa _2$, and *V*. The pseudo-likelihood algorithm then iterates until convergence of these estimates. More details about the pseudo-likelihood method are given in Additional file 1: Section S1.

### BG2 screening step

The BG2 screening step uses a P3D approach based on a baseline model that assumes a linear predictor given in Eq. (2) specialized to contain no SNPs, that is, $g(E(\pmb {y}|\pmb {\beta }_c, \pmb {\alpha }_1,\pmb {\alpha }_2)) = X_c\pmb {\beta }_c+\pmb {\alpha }_1+\pmb {\alpha }_2.$

Our P3D approach keeps $\pmb {\beta }_c$, $\kappa _1$, $\kappa _2$, and *V* fixed at their pseudo-likelihood estimates when performing the Bayesian SMA in the BG2 screening step. Let us denote these estimates by $\widehat{\pmb {\beta }}_c$, ${\widehat{\kappa }}_1$, ${\widehat{\kappa }}_2$, and ${\widehat{V}}$. In addition, our P3D approach keeps the vector of adjusted observations fixed equal to $\pmb {y}^\star$ obtained at the last iteration of the pseudo-likelihood algorithm for the baseline model. Let $H= {\widehat{\kappa }}_1\Sigma + {\widehat{\kappa }}_2 I+ {\widehat{V}}^{-1}$ be the estimated covariance matrix of the adjusted observations $\pmb {y}^\star$. Consider the spectral decomposition of the matrix *H* given by $H=PDP^T$. The matrix *H* is kept fixed for all SNPs in the screening step. Thus, the spectral decomposition of *H*, which has a computational cost of $O(n^3)$, has to be computed only once at the beginning of the screening step.

Let $\pmb {x}_s$ be the vector of covariate values for SNP *s*. Then, the BG2 screening step assumes for each SNP *s*, $s=1,\dots ,p$, that the adjusted observations $\pmb {y}^\star$ can be modeled by the LMM

4 $$\begin{aligned} \pmb {y}^\star= X_c \widehat{\pmb {\beta }}_c + \pmb {x}_s \beta _s+ \pmb {\alpha }_1 + \pmb {\alpha }_2 + {\widehat{V}}^{-1}\pmb {\epsilon }. \end{aligned}$$

Then, the adjusted observations $\pmb {y}^\star$ have an approximate multivariate Gaussian distribution $N(X_c \widehat{\pmb {\beta }}_c + \pmb {x}_s \beta _s, H).$ Let $\widetilde{\pmb {y}} = P^T(\pmb {y}^\star -X_c\widehat{\pmb {\beta }}_c)$ and $\widetilde{\pmb {x}}_s = P^T \pmb {x}_s$. Then, an estimator of $\beta _s$ is ${\widehat{\beta }}_s = (\widetilde{\pmb {x}}_s^T D^{-1} \widetilde{\pmb {x}}_s)^{-1} \widetilde{\pmb {x}}_s^T D^{-1} {\widetilde{y}}$. In addition, the estimator ${\widehat{\beta }}_s$ has approximate distribution $N(\beta _s, \sigma ^2_s)$, where $\sigma ^2_s = var( {\widehat{\beta }}_s) = (\widetilde{\pmb {x}}_s^T D^{-1} \widetilde{\pmb {x}}_s)^{-1}.$

We assign for $\beta _s$ a prior that is a mixture of a Dirac delta function and a nonlocal prior, that is,

$$\begin{aligned} p(\beta _s|\tau ,\pi _0)= \pi _0{\delta _0(\beta _s)}+(1-\pi _0)\frac{\beta _s^2}{n\tau \sigma _s^2}N(\beta _s|0,n\tau \sigma _s^2), \end{aligned}$$

where $\pi _0$ is the probability of the null hypothesis that $\beta _s$ is equal to zero and $\tau >0$ is a scale parameter. Larger values of $\tau$ cause the prior to be more spread out and lead BG2 to focus on identifying SNPs with relatively large regression coefficients. Then, the predictive density of ${\widehat{\beta }}_s$ is

5 $$\begin{aligned} p( {\widehat{\beta }}_s|\tau ,\pi _0)&= \int p( {\widehat{\beta }}_s|\beta _s) p(\beta _s|\tau ,\pi _0) \, d\beta _s \nonumber \\ &= \pi _0N( \widehat{\beta _s}|0,\sigma _s^2)+(1-\pi _0)(2\pi \sigma _s^2)^{-\frac{1}{2}}(n\tau +1)^{-\frac{3}{2}} \nonumber \\{} &\quad \exp \left\{ -\frac{ \widehat{\beta _s^2}}{2\sigma _s^2 (n\tau +1)} \right\} \left[ 1+\frac{n\tau \widehat{\beta _s^2}}{(n\tau +1)\sigma _s^2} \right] . \end{aligned}$$

Based on this predictive density and assuming that ${\widehat{\beta }}_1, \ldots , {\widehat{\beta }}_p$ are approximately conditionally independent given $\pi _0$ and $\tau$, we obtain the approximate likelihood function of $\tau$ and $\pi _0$

6 $$\begin{aligned} L({\widehat{\beta }}_1, \ldots , {\widehat{\beta }}_p| \tau , \pi _0)= & {} \prod _{s=1}^{p} p( {\widehat{\beta }}_s|\tau ,\pi _0). \end{aligned}$$

Let $\pi (\tau )$ and $\pi (\pi _0)$ be the prior densities of $\tau$ and $\pi _0$, respectively. Then, by Bayes Theorem an approximate posterior density for $(\tau ,\pi _0)$ is

7 $$\begin{aligned} \pi (\tau , \pi _0|{\widehat{\beta }}_1, \ldots , {\widehat{\beta }}_p)\propto & {} \pi (\tau )\pi (\pi _0) \prod _{s=1}^{p} p( {\widehat{\beta }}_s|\tau ,\pi _0). \end{aligned}$$

BG2 estimates $\tau$ and $\pi _0$ by maximizing (7) to obtain posterior modes ${\widehat{\tau }}$ and ${\widehat{\pi }}_0$.

We assign a noninformative uniform prior on (0, 1) for $\pi _0$ and consider two prior specifications for $\tau$. The first prior specification is a uniform prior for $\tau$ on $(0,\infty )$. The second prior specification for $\tau$ is an inverse gamma distribution with shape parameter $0.55/0.022 + 1$ and rate parameter 0.55, that is $\tau \sim IG(0.55/0.022+1,0.55)$. This prior specification implies a prior mean for $\tau$ equal to 0.022, which was the value for a fixed $\tau$ recommended by [20] for GWAS studies. In addition, we note that values of $\tau$ that are too small lead to numerical instability. Therefore, our prior specification implies that *a priori* $P(\tau >0.01)=0.999$, stochastically keeping $\tau$ away from 0.

Alternatively, we may fix $\tau$ at pre-specified values [14, 20]. Specifically, in the context of GWAS analysis, [20] suggested fixing $\tau =0.022$ because GWAS effect sizes are generally very small. When $\tau =0.022$, the nonlocal product moment prior (pMOM) prior assigns a probability of 0.01 to the event that a standardized effect size falls in the interval ($-$0.05, 0.05). Thus, in the simulation studies presented in Sect. 4, we also consider fixing $\tau$ at 0.022.

After estimating $\tau$ and $\pi _0$, BG2 takes an Empirical Bayes approach and keep them at their estimates ${\widehat{\tau }}$ and ${\widehat{\pi }}_0$ while using Bayes Theorem to compute the posterior probability that the regression coefficient of SNP s ($s=1,\ldots ,p$) in the screening step is different than zero, that is

8 $$\begin{aligned} P(\beta _s \ne 0 | {\widehat{\beta }}_s, {\widehat{\tau }}, {\widehat{\pi }}_0)= & {} 1 - \frac{{\widehat{\pi }}_0 N( \widehat{\beta _s}|0,\sigma _s^2)}{p( {\widehat{\beta }}_s|{\widehat{\tau }},{\widehat{\pi }}_0)}, \end{aligned}$$

where $p( {\widehat{\beta }}_s|{\widehat{\tau }},{\widehat{\pi }}_0)$ is the predictive density given in Eq. (5) with $\tau ={\widehat{\tau }}$ and $\pi _0={\widehat{\pi }}_0$.

Finally, based on the posterior probabilities computed with Eq. (8), the BG2 screening step uses Bayesian FDR control [8, 9, 27–29] to obtain a list of candidate SNPs while keeping the nominal FDR at 5%. Let us denote the number of SNPs contained in this list of candidate SNPs obtained in the screening step by *k*.

### BG2 model selection step

The BG2 model selection step considers GLMMs with any number of SNPs from the list of *k* candidate SNPs obtained from the BG2 screening step. Thus, the model selection step considers $S=2^k$ possible models. Let $M_m$ be the *m*-th model, $m = 1, \ldots , S$. Let $X_m$ be the matrix of SNPs in model $M_m$, $\pmb {\beta }_m$ be the corresponding vector of regression coefficients, and $p_m$ be the number of SNPs in model $M_m$. Let $X_S$ be the model with all *k* candidate SNPs.

We assume that the *k* candidate SNPs may or may not be in a model according to a sequence of exchangeable Bernoulli trials. Specifically, the prior probability of model $M_m$ is $P(M_m) = {\widehat{\pi }}_0^{k - p_m} (1- {\widehat{\pi }}_0)^{p_m}$ where ${\widehat{\pi }}_0$ is the estimate of the probability of null hypothesis obtained in the screening step. We do this to ensure that the Bayesian control of false discoveries in the BG2 model selection step is as strict as the control of false discoveries in the BG2 screening step.

The BG2 model selection step uses a P3D approach where the baseline model is the full model $M_S$ with linear predictor $g(E(\pmb {y}|\pmb {\alpha }_1,\pmb {\alpha }_2)) = X_c\pmb {\beta }_c+X_{S}\pmb {\beta }_{S}+\pmb {\alpha }_1+\pmb {\alpha }_2.$ The pseudo-likelihood approach then yields estimates $\widehat{\pmb {\beta }}_c$, ${\widehat{\kappa }}_1$ and ${\widehat{\kappa }}_2$, ${\widehat{V}}$, and adjusted observations $\pmb {y}^\star$. We then consider all models $M_m, m=1,\ldots ,S$, where we keep $\pmb {\beta }_c$, $\kappa _1$, $\kappa _2$, and *V* fixed at these estimates. Let $H= {\widehat{\kappa }}_1\Sigma + {\widehat{\kappa }}_2 I+ {\widehat{V}}^{-1}$ and consider the spectral decomposition of the matrix *H* given by $H=PDP^T$. The matrix *H* is kept fixed for all non-baseline models in the model selection step. Thus, even though the spectral decomposition has a computational cost of $O(n^3)$, this decomposition has to be computed only once at the beginning of the model selection step. In addition, following the recommendation of [25], we keep the adjusted observations for all the *S* considered models fixed at the adjusted observations $\pmb {y}^\star$ obtained while fitting the full model.

Therefore, under model $M_m$ and with the P3D approach, the adjusted observations $\pmb {y}^\star$ follow the approximate distribution $N \left( X_c \widehat{\pmb {\beta }}_c + X_m \pmb {\beta }_m, H \right) .$ In addition, let $\widetilde{\pmb {y}} = P^T(\pmb {y}^\star -X_c\widehat{\pmb {\beta }}_c)$ and ${\widetilde{X}}_m = P^TX_m$. Then, we can rewrite the LMM as $\widetilde{\pmb {y}}|\pmb {\beta }_m {\mathop {\sim }\limits ^{a}} N({\widetilde{X}}_m \pmb {\beta }_m, D)$, where ${\mathop {\sim }\limits ^{a}}$ denotes “approximately distributed as.” Because *D* is a diagonal matrix, computations for this latter model are very fast.

We propose a novel nonlocal prior for GLMMs. Specifically, we propose a prior density that is the product of a multivariate Gaussian density and the product of the square of each element of the vector of regression coefficients $\beta _m$. In this multivariate Gaussian density, the covariance matrix is $\tau n (X_m^T H^{-1} X_m)^{-1}$. Using the spectral decomposition of the matrix *H*, the prior we propose for $\pmb {\beta }_m$ is

9 $$\begin{aligned} { \pi }(\pmb {\beta }_m|M_m)&= d_m (2\pi )^{- p_m/2}( {\widehat{\tau }} n)^{-3 p_m/2}|{\widetilde{X}}_m^T D^{-1} {\widetilde{X}}_m |^{\frac{3}{2}} \nonumber \\{} &\quad \exp \left[ -\frac{1}{2 {\widehat{\tau }} n}\pmb {\beta }_m^T {\widetilde{X}}_m^T D^{-1} {\widetilde{X}}_m \pmb {\beta }_m\right] \prod _{i=1}^{ p_m} \beta _{mi}^2, \end{aligned}$$

where $d_m$ is a normalizing constant.

Let $C_m = {\widetilde{X}}_m^T D^{-1} {\widetilde{X}}_m(1+({\widehat{\tau }} n)^{-1})$, $\widetilde{\pmb {\beta }}_m = C_m^{-1}{\widetilde{X}}_m^T D^{-1} \widetilde{\pmb {y}}$, and $R_m \ = \ \widetilde{\pmb {y}}^T D^{-1} (D-{\widetilde{X}}_m C_m^{-1} {\widetilde{X}}_m^T) D^{-1}\widetilde{\pmb {y}} \ = \ \widetilde{\pmb {y}}^T D^{-1} \widetilde{\pmb {y}}- \widetilde{\pmb {y}}^T D^{-1} {\widetilde{X}}_m\widetilde{\pmb {\beta }}_m.$ Then, the marginal density of the adjusted observations $\widetilde{\pmb {y}}$ conditional on model $M_m$ is

10 $$\begin{aligned} m(\widetilde{\pmb {y}}|M_m)&= \int N(\widetilde{\pmb {y}}|{\widetilde{X}}_m\pmb {\beta }_m,D)\pi (\pmb {\beta }_m|M_m) \, d\pmb {\beta }_m \nonumber \\ &= (2 \pi )^{-\frac{n}{2}} |D|^{-\frac{1}{2}}(1+ {\widehat{\tau }} n)^{- p_m/2} \nonumber \\{} & {} \exp \left( -\frac{R_m}{2}\right) \frac{E_2\left( \prod _{i=1}^{ p_m}\beta _{mi}^2 \right) }{E_1\left( \prod _{i=1}^{ p_m}\beta _{mi}^2 \right) }, \end{aligned}$$

where $E_1\left( \prod _{i=1}^{ p_m}\beta _{mi}^2 \right)$ is the expected value with respect to $N(\pmb {0},(1+ {\widehat{\tau }} n) C_m^{-1} )$ and $E_2\left( \prod _{i=1}^{ p_m}\beta _{mi}^2 \right)$ is the expected value with respect to $N(\widetilde{\pmb {\beta }}_m, C_m^{-1})$. To approximate $E_1\left( \prod _{i=1}^{p_m}\beta _{mi}^2 \right)$ and $E_2\left( \prod _{i=1}^{p_m}\beta _{mi}^2 \right)$, we simulate 2000 samples from $N(\widetilde{\pmb {\beta }}_m, C_m^{-1})$, denoted as $\pmb {\beta }_{2m}^{(j)}, \ j=1,\dots , 2000$. We compute $\sum _{j=1}^{2000}(\prod _{i=1}^{p_m}\beta _{2m i}^{2(j)})/2000$ as an approximation to $E_2\left( \prod _{i=1}^{p_m}\beta _{mi}^2 \right)$. Let $\pmb {\beta }_{1\,m}^{(j)} =(1+ {\widehat{\tau }} n)^{\frac{1}{2}}(\pmb {\beta }_{2\,m}^{(j)}-\widetilde{\pmb {\beta }}_m), \ j=1,\dots , 2000$. Finally, we compute $\sum _{j=1}^{2000}(\prod _{i=1}^{p_m}\beta _{1m i }^{2(j)})/2000$ as an approximation to $E_1\left( \prod _{i=1}^{p_m}\beta _{mi}^2 \right)$.

Then, the posterior probability of model $M_m$ is

11 $$\begin{aligned} P(M_m|\widetilde{\pmb {y}})\propto & {} P(M_m) m(\widetilde{\pmb {y}}|M_m). \end{aligned}$$

Note that the posterior distribution of the vector of regression coefficients is multimodal. BG2 deals with this multimodality without any difficulties. In the screening step, when $\beta _s$ is a scalar, we compute the posterior probability of $\beta _s \ne 0$ using Eqs. (5) and (8). In the model selection step, when $\pmb {\beta }_m$ is a vector of coefficients, we compute the posterior probability of model $M_m$ using Eqs. (10) and (11).

If the number of candidate covariates *k* is small ($k<16$), we compute the posterior probabilities for all $2^{k}$ candidate models and select the highest posterior probability model as the best model. If the number of candidate covariates is large, we use a genetic algorithm from the R package GA [30] to search for the highest posterior probability model.

## Simulation studies

We have performed simulation studies to compare our nonlocal-prior-based BG2 method versus SMA for binary data and count data. Specifically, we consider single marker analysis with Bonferroni correction with nominal FDR set to 0.05. To assess the performance of our methods, in these simulation studies we use genotype SNP data from humans and from A. Thaliana. These are the same genotype data used in the case studies we present in Sect. 5. We use four criteria to compare the competing methods: true positives (TP), false positives (FP), false discovery rate (FDR) and F1 score. Within each simulation study, for each method we compute the average TP, FP, FDR and F1 over 100 simulated datasets. We use a buffer to define what is a true positive and a false positive. Following [21], if one or more detected SNPs are adjacent (within 5000 base pairs) to a same causal SNP, that is counted as a true positive. In addition, each detected SNP not adjacent to a causal SNP is counted as a false positive.

### Binary data

We simulate binary GWAS data using genotype information from the Study of Addiction: Genetics and Environment (SAGE) which is part of the National Human Genome Research Institute’s Gene Environment Association Study Initiative [Database for Genotypes and Phenotypes (dbGaP) study accession phs000092.v1.p1]. Specifically, we use genotype information from 2,772 European Americans in a total of 800,000 SNPs with minor allele frequency (MAF) larger than 0.01.

From these 800,000 SNPs, we selected 20 evenly spaced SNPs to be the causal SNPs. We set the regression coefficients for 5 of these causal SNPs to 0.2, and for 5 other causal SNPs to $-$0.2. In addition, the regression coefficients for the other 10 causal SNPs have the same value $\beta$, but that value varies in six settings: 0.2, 0.3, 0.4, 0.5, 0.7 and 1. Further, we set the intercept at $\beta _0=-0.5$. Furthermore, the variance component $\kappa$ of the kinship random effects $\pmb {\alpha }$ is set to 0.15. Thus, the binary phenotype data are simulated from a Bernoulli GLMM with logistic link function and linear predictor $\beta _0 + \sum _{i=1}^{10} \beta x_{ij} + \sum _{i=11}^{15} 0.2x_{ij} + \sum _{i=16}^{20} (-0.2)x_{ij} + \alpha _i,$ with $\pmb {\alpha } \sim N(\pmb {0}, \kappa \Sigma )$ where $\Sigma$ is the kinship matrix.

**Fig. 1:**
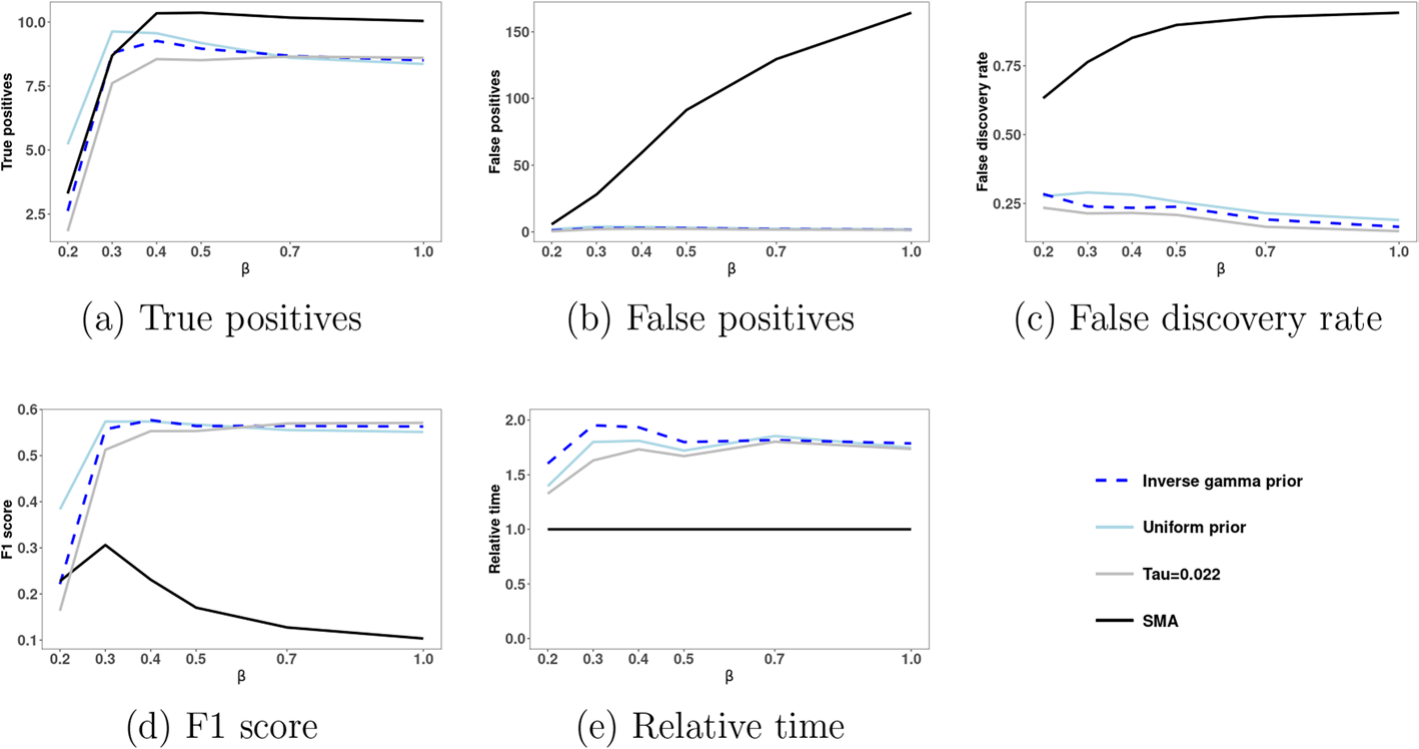
SNP search performance of BG2 and SMA methods for simulated binary data

Figure 1 shows for binary data the performance of our BG2 method with three different ways to choose the parameter $\tau$, as well as the performance of the SMA method. These performances in terms of TP, FP, FDR, and F1 averaged over 100 datasets for each setting are plotted as functions of the varying regression coefficient $\beta$. In addition, Fig. 1 shows the computational time. Additional file 1: Figure S2 show boxplots of TP, FP, FDR, and F1. Our BG2 methods take twice as long as SMA, which is to be expected since SMA has only a screening step whereas BG2 has a screening step and a model selection step. Among the three ways considered to choose $\tau$ for BG2, estimating $\tau$ based on a uniform prior provides higher F1 scores for smaller values of $\beta$, and provides comparable F1 scores for larger values of $\beta$. In addition, when compared to SMA, BG2 with uniform prior provides larger average number of true positives TP than when $\beta$ is small, and a smaller TP when $\beta$ is large. However, BG2 with uniform prior leads to a much smaller average number of false positives than SMA. As a result, when compared to SMA, for all considered values of the regression coefficient $\beta$, BG2 with uniform prior has much larger F1.

Finally, we have tested the robustness of BG2 to the case of binary GWAS data with no causal SNPs. Specifically, we have simulated 100 datasets with binary GWAS data from a Bernoulli GLMM with logit link function and linear predictor $\beta _0 + \alpha _i$. While BG2 with any of the ways to choose $\tau$ does not yield any false positive for 100 simulated datasets, SMA has an average of 0.06 false positives. Therefore, BG2 performs better than SMA for binary GWAS data and is robust to the case when there are no causal SNPs.

### Count data

We simulate count GWAS data using genotype information from The Arabidopsis Information Resource (TAIR9) (https://www.arabidopsis.org/). This simulation study is based on a case study on root-like structures in *A. Thaliana* that we present in Sect. 5.3.

Specifically, we use 188,980 SNPs with MAF>0.01 from 152 ecotypes of *A. Thaliana*. This simulation study assumes 10 causal SNPs evenly located among all available SNPs. Of these 10 causal SNPs, 5 causal SNPs have fixed coefficients equal to 0.2, and the other 5 causal SNPs have the same coefficient $\beta$ which varies in eight settings: 0.1, 0.2, 0.3, 0.4, 0.5, 0.6, 0.7 and 1. In addition, we set the intercept $\beta _0$ equal to 1. Further, we assume that there are two random effects: a kinship random effect $\pmb {\alpha }_1$ with variance component $\kappa _1$ equal to 1; and an overdispersion random effect $\pmb {\alpha }_2$ with variance component $\kappa _2$ equal to 0.3, which is close to the estimate obtained in the case study presented in Sect. 5.3. Let $r_i$ be the number of replicates of ecotype *i*. Because in the case study most ecotypes have 12 replicates, in this simulation study we assume that all ecotypes have 12 replicates. In addition, the phenotype $y_i$ for ecotype *i* is the total number of root-like structures of the $r_i$ replicates. These phenotype count data are sampled from a Poisson GLMM with logarithm link function and linear predictor $\log (r_i) + \beta _0 + \sum _{i=1}^5 \beta x_{ij} +\sum _{i=6}^{10} 0.2 x_{ij} +\alpha _{1i} +\alpha _{2i}$.

**Fig. 2:**
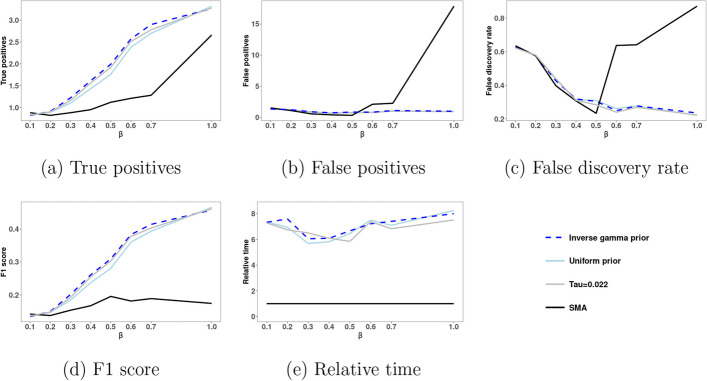
SNP search performance of BG2 and SMA methods for simulated count data

Figure 2 shows for count data the performance of our BG2 method as well as the performance of the SMA method. These performances are averaged over 100 simulated datasets for each setting and plotted as functions of the varying regression coefficient $\beta$. In addition, Fig. 2 shows the computational time. Additional file 1: Figure S3 show boxplots of TP, FP, FDR, and F1. Our BG2 methods take about eight times longer than SMA, but they still provide results in a feasible amount of time. Among the three ways considered to choose $\tau$ for BG2, estimating $\tau$ based on an inverse gamma prior provides larger average number of true positives and about the same FDR level. As a result, when compared to the other ways to choose $\tau$, estimating $\tau$ based on an inverse gamma prior has higher F1 scores for most considered values of $\beta$. In addition, when compared to SMA, BG2 with an inverse gamma prior provides larger average number of true positives TP for most considered values of $\beta$. Further, BG2 with inverse gamma prior has about the same FDR level as SMA for $\beta \le 0.5$ and a much smaller FDR level for $\beta > 0.5$. As a result, while BG2 with an inverse gamma prior has comparable F1 to SMA for small values of $\beta$, the F1 of BG2 with an inverse gamma prior becomes much larger than the F1 of SMA as $\beta$ increases.

In addition, we have tested the robustness of BG2 to the case of count GWAS data with no causal SNPs. Specifically, we have simulated 100 datasets with count GWAS data from a Poisson GLMM with logarithm link function and linear predictor $\beta _0 + \alpha _{1i} +\alpha _{2i}$. The average number of false positives for all considered methods is 0. Thus, both SMA and BG2 methods perform well in the case of count GWAS data with no causal SNPs.

### Choice of prior for $\tau$

Choice of priors is an important part of the implementation of Bayesian methods. To obtain more information about the impact of prior choice on the results of BG2 implementations, we have expanded our simulation studies presented in Sects. 4.1 and 4.2. Specifically, we have performed two additional simulation studies: one that uses count data simulated with human genome and another one that uses binary data simulated with A. Thaliana genome. Unfortunately, the simulated binary datasets simulated with A. Thaliana genome did not contain enough information for SNPs to be detected by SMA or BG2. Additional file 1: Figure S1 presents the results for count data based on human genome data. Similarly to the results from Sects. 4.1 and 4.2, any of the implementations of BG2 perform much better than SMA in terms of FDR and F1. In addition, for count data based on human genome data, the three implementations of BG2 provide similar results. Therefore, from the results of the simulation studies, there is no prior choice that dominates the other prior choices.

Another important consideration is that the performance of the priors will vary for different GWAS datasets. And, of course, BG2 is to be used by scientists who are not Bayesian statisticians – thus, ideally there should be a default prior that would be safe to use with any GWAS dataset. One such class of priors is the class of non-informative priors [31–34] that impart little or no information in the analysis. The uniform prior is not concentrated around any particular value of $\tau$ and, in this sense, is non-informative. In addition, in the simulation studies considered here, BG2 with the uniform prior for $\tau$ performed similarly or better than BG2 with other prior choices for $\tau$. Therefore, in the implementation of BG2 we recommend the uniform prior as a default choice for $\tau$.

## Case studies

To illustrate the usefulness and flexibility of the nonlocal-prior-based BG2 method, this section presents three case studies on cocaine dependence, alcohol consumption, and number of root-like structures in *A. Thaliana*.

### Maximum number of alcoholic drinks

The Collaborative Study on the Genetics of Alcoholism (COGA) [35] was a large-scale family study that had as primary objective to identify genes related to alcohol dependence. Here, we consider as the response variable the maximum number of alcoholic drinks consumed in 24 h. We analyze data on 2759 European Americans considering 846,076 SNPs with MAF>0.01 and with less than 5% missing. To perform this analysis, we use the Poisson GLMM for count data considered in Sect. 4.2. In our analysis, the 846,076 SNPs are possible regressors. Our Poisson GLMM accounts for genetic structure among 2759 subjects by including a vector of kinship random effects, and allows for extra-Poisson variability with a vector of overdispersion random effects.

While SMA detected 10 SNPs, BG2 detected only one SNP. More specifically, the screening step of BG2 identified 10 candidate SNPs which were then given to the BG2 model selection step. The BG2 model selection step then identified one SNP. Likelihood ratio tests indicate that the identified SNPs do not violate the hypothesis of Hardy-Weinberg equilibrium. While we cannot be sure about which of these SNPs are false positives, the simulation studies in Sect. 4 show that SMA tends to have a much higher FDR than BG2. Thus, in this case study the nonlocal-prior-based BG2 method provides a list of SNPs for further investigation that is much more focused. The SNP detected by BG2 is located in the protein-coding gene PTGER4 on chromosome 5. The protein encoded by PTGER4 is a receptor for prostaglandin E2 (PGE2). An increase in PGE2 is part of the inflammatory response to alcohol consumption, and the use of the PGE2-inhibitor tolfenamic acid significantly reduces the severity of several hangover symptoms [36].

### Cocaine dependence

In this case study, we analyze the association between cocaine dependence and single nucleotide polymorphisms (SNPs). We analyze data from the Family Study of Cocaine Dependence (FSCD) [37], which was part of the Study of Addiction: Genetics and Environment. Specifically, we analyze data on 2,767 European Americans considering 846,076 SNPs with MAF>0.01 and with less than 5% missing. The response variable is whether or not the subject is addicted to cocaine. To perform this analysis, we use the model for binary data considered in Sect. 4.1. Because males and females seem to have different behaviors with respect to cocaine use, we include sex as a control covariate. All 846,076 SNPs are possible regressors. In addition, to account for the genetic structure among the 2767 subjects, our Bernoulli GLMM has a vector of kinship random effects.

BG2 detects one SNP, which is located in the protein-coding gene ABCC8 on chromosome 11. In this case study, the screening step of BG2 identified 1 candidate SNP which was then selected in the model selection step. For this dataset, SMA only detects the same SNP. A likelihood ratio test indicates that the identified SNP does not violate the hypothesis of Hardy-Weinberg equilibrium. The protein encoded by this gene is a member of the superfamily of ATP-binding cassette (ABC) proteins which transport various molecules across extra-cellular and intra-cellular membranes. In addition, a quantitative transcriptomics analysis (RNA-Seq) has shown that this gene is overexpressed in the brain [38]. Further, cocaine increases expression of ABCC1 (another gene that encodes an ABC protein) in mice [39]. Finally, ABCC1-siRNA (a silencer of ABCC1) blocks cocaine-induced place preference in mice [39].

### Root-like structures in *A. Thaliana*

To illustrate the application of our method to count data, we analyze data from a study of plant regeneration from root explants of the selfing species *A. Thaliana* [40]. Specifically, we consider as response variable the number of root-like structures. We note that [40] applied a square root transformation to analyze this count phenotype variable. In contrast, we use the Poisson GLMM with overdispersion considered in Sect. 4.2 to analyze the original count data. Our model contains a vector of kinship random effects to account for the correlation among individuals and a vector of overdispersion random effects. We focus on the number of root-like structures after 21 days in which seedlings are under warm white light at 21$^o$C following a 14/10 h light/dark regime. There are 188,980 SNPs for 152 ecotypes, with 12 biological replicates per ecotype, from TAIR9 with MAF>0.01.

BG2 detects 3 SNPs. More specifically, the screening step of BG2 screened 5 candidate SNPs and then the BG2 model selection step identified 3 of these SNPs. For this dataset, SMA detects the same 3 SNPs. These 3 SNPs are expressed in the root and are located in protein-coding genes AT1G20090, AT1G20100 and AT1G20720. Specifically, AT1G20100 encodes a DNA ligase-like protein involved in the regulation of metabolic processes. In addition, gene AT1G20720 encodes a RAD3-like DNA binding helicase protein that acts in the repair of double-strand breaks in DNA, and in nucleotide-excision repair. Finally, AT1G20090 encodes a ROP2 protein which is known to effect root hair initiation and tip growth [41].

## Discussion

We have proposed BG2, a two-stage Bayesian SNP detection method for non-Gaussian GWAS data. BG2 uses a GLMM framework that includes kinship random effects and overdispersion random effects. BG2 has two steps: a screening step and a model selection step. The screening step performs a Bayesian SMA that selects a set of candidate SNPs. The model selection step then considers all possible GLMMs based on this set of candidate SNPs. To speed up computations, we develop a pseudo likelihood approach combined with P3D. Further, we develop a novel class of nonlocal priors for the regression coefficients specially tailored for GLMMs. Simulation studies show that, for both binary and count GWAS data, BG2 is much better than SMA in terms of FDR and F1.

The simulation studies show that, when compared to SMA, BG2 has a much lower FDR. Of course, there are some combinations of parameters for which SMA and BG2 provide similar results, and that is what seems to have happened in Sects. 5.2 and 5.3. However, in some applications BG2 provides a much smaller number of false discoveries than SMA, and that is what seems to have happened in the case study presented in Sect. 5.1. Therefore, when compared to SMA, BG2 is more robust and precise.

A relevant question is how sensitive to the choice of prior is the performance of BG2. We considered here implementations of BG2 with three different choices of priors. The simulation studies presented in this paper and in the additional file have shown that BG2 implementations with each of these three different choices of priors have similar performance. As a matter of fact, BG2 with the different choices of priors considered here provide the same results for the three case studies. Therefore, the performance of BG2 seems to be relatively robust to the choice of priors.

While we have chosen to implement BG2 with a pseudo-likelihood approach and a genetic algorithm to explore the model space for the analysis of non-Gaussian GWAS data, we acknowledge that other approaches may be possible. For example, instead of the pseudo-likelihood approach, researchers may consider variational inference approaches [42, 43]. In addition, instead of combining the pseudo-likelihood approach and a genetic algorithm, researchers may consider implementing a parallel tempering approach [44] to perform estimation and model selection at the same time. However, we think that such parallel tempering approach may not be computationally feasible for Bayesian ultra-high dimensional variable selection in GLMMs applied to non-Gaussian GWAS.

There are several possible avenues for future research. One promising research direction is to adapt BG2 for application to biobank scale data. Another possible research direction is to implement BG2 with an iterative procedure that would allow smaller effect sizes to be detected. Finally, another possible research avenue is to develop BG2 for GWAS analysis when the phenotype is survival time.

## Conclusion

We propose BG2, a novel two-stage Bayesian approach for non-Gaussian GWAS data. Compared to SMA, BG2 provides a much lower FDR, is more precise and robust. BG2 is implemented in the R package BG2 that is available on BioConductor at https://bioconductor.org/packages/release/bioc/html/BG2.html.

### Supplementary Information

**Additional file 1**. Supplementary Material for BG2: Bayesian variable selection in generalized linear mixed models with nonlocal priors for non-Gaussian GWAS data.

## Data Availability

BG2 is implemented in the R package BG2 that is available on BioConductor at https://bioconductor.org/packages/release/bioc/html/BG2.html. In addition, the case study with A. Thaliana can be reproduced using the information and R code available at https://marf-at-vt.github.io/BG2-CaseStudy.html. The A. Thaliana phenotype data and genotype data are available from the following sources: A. Thaliana phenotype data available at https://arapheno.1001genomes.org; A. Thaliana genotype dataset available from R package qtcat.data (https://rdrr.io/github/QTCAT/qtcat.data/). Genotype and phenotype data for alcohol and cocaine use disorder in humans is available from the NIH dbGap website: https://www.ncbi.nlm.nih.gov/gap/, the accession number is phs000092.v1.p1.

## Abbreviations

BG2: Bayesian GLMMs for GWAS
LMM: Linear mixed model
GLMM: Generalized linear mixed model
SMA: Single marker analysis
GWAS: Genome-wide association studies
SNP: Single nucleotide polymorphism
TP: True positive
FP: False positive
FDR: False discovery rate
GA: Genetic algorithm
MAF: Minor allele frequency
P3D: Population parameters previously determined
MCMC: Markov chain Monte Carlo
