# Supplementary material for: BG2: Bayesian variable selection in generalized linear mixed models with nonlocal priors for non-Gaussian GWAS data
Source: BMC Bioinformatics. 2023 Sep 15;24:343. doi: 10.1186/s12859-023-05468-w (PMC10503129; doi:10.1186/s12859-023-05468-w)
Supplement: Supplementary file 1 — Additional file 1. Supplementary Material for BG2: Bayesian variable selection in generalized linear mixed models with nonlocal priors for non-Gaussian GWAS data. [file 12859_2023_5468_MOESM1_ESM.pdf]

Supplementary Material for BG2: Bayesian variable selection in generalized linear mixed models with nonlocal priors for non-Gaussian GWAS data, by Xu, Williams and Ferreira

S1. The pseudo-likelihood approach

In this section, we explain in detail the pseudo-likelihood method for the analysis of non-Gaussian GWAS data. The main point is that our approach performs different estimation procedures for baseline models and for non-baseline models. In what follows, Subsection S1.1 provides details on the estimation procedure for baseline models and Subsection S1.2 provides details on the estimation procedure for non-baseline models.

S1.1. The pseudo-likelihood approach for the baseline models

For fitting of baseline models, our approach uses an iterative pseudo-likelihood algorithm. The algorithm starts with some initial estimates of the parameters $\boldsymbol{\beta}_c$, $\boldsymbol{\beta}_s$, $\boldsymbol{\alpha}_1$, and $\boldsymbol{\alpha}_2$, typically by assuming a generalized linear model. Denote by $\widehat{\boldsymbol{\beta}}_c$, $\widehat{\boldsymbol{\beta}}_s$, $\widehat{\boldsymbol{\alpha}}_1$, and $\widehat{\boldsymbol{\alpha}}_2$ the current estimates of $\boldsymbol{\beta}_c$, $\boldsymbol{\beta}_s$, $\boldsymbol{\alpha}_1$, and $\boldsymbol{\alpha}_2$, respectively. The pseudo-likelihood method is based on a first-order Taylor expansion. Write the vector of observations $\boldsymbol{y}$ as the sum of its mean vector $\boldsymbol{\mu}$ and an error vector $\boldsymbol{\epsilon}$, that is $\boldsymbol{y} = \boldsymbol{\mu} + \boldsymbol{\epsilon}$. Here, the mean vector is $\boldsymbol{\mu} = B'(\boldsymbol{\eta}) = m(\boldsymbol{\eta})$ and the covariance matrix of the error vector is the diagonal matrix $V = diag(v(\boldsymbol{\eta}))$, where $v(\boldsymbol{\eta}) = (v_1, \dots, v_n)' = (B''(\eta_1), \dots, B''(\eta_n))'$. Let X_c be the matrix of control covariates and X_s be the matrix of SNPs. Expand the mean vector using a first-order Taylor expansion about $\widehat{\boldsymbol{\beta}}_c$, $\widehat{\boldsymbol{\beta}}_s$, $\widehat{\boldsymbol{\alpha}}_1$, and $\widehat{\boldsymbol{\alpha}}_2$. Then,

$$
\begin{aligned}
\boldsymbol{y} &= \boldsymbol{\mu} + \boldsymbol{\epsilon} \\
&= B'(\boldsymbol{\eta}) + \boldsymbol{\epsilon} \\
&\approx B'(\widehat{\boldsymbol{\eta}}) + B''(\widehat{\boldsymbol{\eta}})[X_c(\boldsymbol{\beta}_c - \widehat{\boldsymbol{\beta}}_c) + X_s(\boldsymbol{\beta}_s - \widehat{\boldsymbol{\beta}}_s) + \boldsymbol{\alpha}_1 - \widehat{\boldsymbol{\alpha}}_1 + \boldsymbol{\alpha}_2 - \widehat{\boldsymbol{\alpha}}_2] + \boldsymbol{\epsilon}
\end{aligned}
$$

$$
\approx B'(X_c\widehat{\boldsymbol{\beta}}_c + X_s\widehat{\boldsymbol{\beta}}_s + \widehat{\boldsymbol{\alpha}}_1 + \widehat{\boldsymbol{\alpha}}_2) + B''(X_c\widehat{\boldsymbol{\beta}}_c + X_s\widehat{\boldsymbol{\beta}}_s + \widehat{\boldsymbol{\alpha}}_1 + \widehat{\boldsymbol{\alpha}}_2)[X_c(\boldsymbol{\beta}_c - \widehat{\boldsymbol{\beta}}_c) + X_s(\boldsymbol{\beta}_s - \widehat{\boldsymbol{\beta}}_s) + \boldsymbol{\alpha}_1 - \widehat{\boldsymbol{\alpha}}_1 + \boldsymbol{\alpha}_2 - \widehat{\boldsymbol{\alpha}}_2] + \boldsymbol{\epsilon}. \tag{S.1}
$$

where $\widehat{\boldsymbol{\beta}}_c$, $\widehat{\boldsymbol{\beta}}_s$, $\widehat{\boldsymbol{\alpha}}_1$, and $\widehat{\boldsymbol{\alpha}}_2$ are the current estimates of $\boldsymbol{\beta}_c$, $\boldsymbol{\beta}_s$, $\boldsymbol{\alpha}_1$, and $\boldsymbol{\alpha}_2$, respectively.

Denote the current estimate of the mean vector $\boldsymbol{\mu}$ evaluated at $\widehat{\boldsymbol{\beta}}_c$, $\widehat{\boldsymbol{\beta}}_s$, $\widehat{\boldsymbol{\alpha}}_1$ and $\widehat{\boldsymbol{\alpha}}_2$ by $\widehat{\boldsymbol{\mu}}$. That is,

$$
\widehat{\boldsymbol{\mu}} = m(X_c\widehat{\boldsymbol{\beta}}_c + X_s\widehat{\boldsymbol{\beta}}_s + \widehat{\boldsymbol{\alpha}}_1 + \widehat{\boldsymbol{\alpha}}_2).
$$

In addition, denote the current estimate of the covariance matrix of $\boldsymbol{\epsilon}$ evaluated at $\widehat{\boldsymbol{\beta}}_c$, $\widehat{\boldsymbol{\beta}}_s$, $\widehat{\boldsymbol{\alpha}}_1$ and $\widehat{\boldsymbol{\alpha}}_2$ by $\widehat{V}$, that is

$$
\widehat{V} = diag[v(X_c\widehat{\boldsymbol{\beta}}_c + X_s\widehat{\boldsymbol{\beta}}_s + \widehat{\boldsymbol{\alpha}}_1 + \widehat{\boldsymbol{\alpha}}_2)].
$$

Reorganize Equation (S.1) by moving $\widehat{\boldsymbol{\mu}}$ to the left side, pre-multiplying $\widehat{V}^{-1}$ and then moving $X_c\widehat{\boldsymbol{\beta}}_c$, $X_s\widehat{\boldsymbol{\beta}}_s$, $\widehat{\boldsymbol{\alpha}}_1$ and $\widehat{\boldsymbol{\alpha}}_2$ to the left side. Let $\boldsymbol{y}^\star$ be equal to the left side of the resulting equation, that is

$$\boldsymbol{y}^\star = \widehat{V}^{-1}(\boldsymbol{y} - \widehat{\boldsymbol{\mu}}) + X_c\widehat{\boldsymbol{\beta}}_c + X_s\widehat{\boldsymbol{\beta}}_s + \widehat{\boldsymbol{\alpha}}_1 + \widehat{\boldsymbol{\alpha}}_2.$$

The vector $\boldsymbol{y}^\star$ is known as the vector of adjusted observations, which is computed as a function of the current estimates of fixed effects $\widehat{\boldsymbol{\beta}}_c$ and $\widehat{\boldsymbol{\beta}}_s$, and random effects $\widehat{\boldsymbol{\alpha}}_1$ and $\widehat{\boldsymbol{\alpha}}_2$. The right side of the resulting equation is $X_c\boldsymbol{\beta}_c + X_s\boldsymbol{\beta}_s + \boldsymbol{\alpha}_1 + \boldsymbol{\alpha}_2 + \widehat{V}^{-1}\boldsymbol{\epsilon}$. Hence, we obtain the following approximate model for the adjusted observations

$$\boldsymbol{y}^\star \approx X_c\boldsymbol{\beta}_c + X_s\boldsymbol{\beta}_s + \boldsymbol{\alpha}_1 + \boldsymbol{\alpha}_2 + \widehat{V}^{-1}\boldsymbol{\epsilon}.$$

Further, assuming that $\widehat{V}^{-1}V\widehat{V}^{-1} \approx \widehat{V}^{-1}$ and applying properties of expectation and variance, we get

$$\begin{aligned} E(\boldsymbol{y}^\star) &= X_c\boldsymbol{\beta}_c + X_s\boldsymbol{\beta}_s, \\ Var(\boldsymbol{y}^\star) &\approx \kappa_1\Sigma + \kappa_2 I + \widehat{V}^{-1}. \end{aligned}$$

If we further assume that $\boldsymbol{\epsilon}$ has an approximate normal distribution, then

$$\boldsymbol{y}^\star \sim N\left(X_c\boldsymbol{\beta}_c + X_s\boldsymbol{\beta}_s, \kappa_1\Sigma + \kappa_2 I + \widehat{V}^{-1}\right).$$

As we explain below, estimates of $\boldsymbol{\beta}_c$, $\boldsymbol{\beta}_s$, $\boldsymbol{\alpha}_1$, $\boldsymbol{\alpha}_2$, κ_1 and κ_2 are updated iteratively. After convergence, we have the final vector of adjusted observations $\boldsymbol{y}^\star$ and the approximate LMM

$$\begin{aligned} \boldsymbol{y}^\star &\approx X_c\boldsymbol{\beta}_c + X_s\boldsymbol{\beta}_s + \boldsymbol{\alpha}_1 + \boldsymbol{\alpha}_2 + \widehat{V}^{-1}\boldsymbol{\epsilon}, \\ \boldsymbol{\alpha}_1 &\sim N(\mathbf{0}, \kappa_1\Sigma), \\ \boldsymbol{\alpha}_2 &\sim N(\mathbf{0}, \kappa_2 I), \\ \boldsymbol{\epsilon} &\sim N(\mathbf{0}, V). \end{aligned}$$

The closed form of the likelihood function with respect to the unknown parameters is

$$\begin{aligned} L(\boldsymbol{\beta}_c, \boldsymbol{\beta}_s, \kappa_1, \kappa_2 | \boldsymbol{y}^\star) &= (2\pi)^{-\frac{n}{2}} \left|\kappa_1\Sigma + \kappa_2 I + \widehat{V}^{-1}\right|^{-\frac{1}{2}} \\ &\quad \exp\left\{-\frac{1}{2}(\boldsymbol{y}^\star - X_c\boldsymbol{\beta}_c - X_s\boldsymbol{\beta}_s)^T(\kappa_1\Sigma + \kappa_2 I + \widehat{V}^{-1})^{-}(\boldsymbol{y}^\star - X_c\boldsymbol{\beta}_c - X_s\boldsymbol{\beta}_s)\right\}. \end{aligned}$$

Let $\widehat{\kappa}_1$ and $\widehat{\kappa}_2$ be current estimates of the variance components. We update $\widehat{\boldsymbol{\beta}}_c$ and $\widehat{\boldsymbol{\beta}}_s$ with the conditional posterior mean of $\boldsymbol{\beta} = (\boldsymbol{\beta}_c', \boldsymbol{\beta}_s')'$, given by $\widehat{\boldsymbol{\beta}} = (X^T\widehat{H}^{-1}X)^{-1}X^T\widehat{H}^{-1}\boldsymbol{y}^\star$, where $X = (X_c, X_s)$ and $\widehat{H} = \widehat{\kappa}_1\Sigma + \widehat{\kappa_2}I + \widehat{V}^{-1}$. Given current estimates $\widehat{\boldsymbol{\beta}}_c$ and $\widehat{\boldsymbol{\beta}}_s$, we use the conditional posterior mean to update the estimates of $\boldsymbol{\alpha}_1$ and $\boldsymbol{\alpha}_2$, that is

$$\begin{aligned} \widehat{\boldsymbol{\alpha}}_1 &= E\left(\boldsymbol{\alpha}_1 | \boldsymbol{y}^\star\right) \\ &= \widehat{\kappa}_1\Sigma\widehat{H}^{-1}(\boldsymbol{y}^\star - X_c\widehat{\boldsymbol{\beta}}_c - X_s\widehat{\boldsymbol{\beta}}_s), \\ \widehat{\boldsymbol{\alpha}}_2 &= \widehat{\kappa}_2\widehat{H}^{-1}(\boldsymbol{y}^\star - X_c\widehat{\boldsymbol{\beta}}_c - X_s\widehat{\boldsymbol{\beta}}_s). \end{aligned} \tag{1}$$

Algorithm 1 Pseudo-likelihood approach for baseline models

procedure PSEUDO LIKELIHOOD($\boldsymbol{y}, X_c, X_s$)
  Initial values: $\boldsymbol{\beta}_c^{(0)}, \boldsymbol{\beta}_s^{(0)}$ = estimates from GLM, $\boldsymbol{\alpha}_1^{(0)}, \boldsymbol{\alpha}_s^{(0)} = \mathbf{0}$, $\kappa_1^{(0)}, \kappa_2^{(0)} = 0$.
  Calculate $\boldsymbol{\mu}^{(0)}$, $V^{(0)}$, $H^{(0)}$ and $\boldsymbol{y}^{\star(0)}$.
  while $\boldsymbol{\beta}_c$, $\boldsymbol{\beta}_s$, κ_1 and κ_2 not converge **do**
    $\boldsymbol{\beta}_c^{(t)} = (X_c^T H^{(t-1)-} X_c)^{-} X_c^T H^{(t-1)-} \boldsymbol{y}^{\star(t-1)}$
    $\boldsymbol{\beta}_s^{(t)} = (X_s^T H^{(t-1)-} X_s)^{-} X_s^T H^{(t-1)-} \boldsymbol{y}^{\star(t-1)}$
    $\boldsymbol{\alpha}_1^{(t)} = \kappa_1^{(t-1)} \Sigma H^{(t-1)-} (\boldsymbol{y}^{\star(t-1)} - X_c \boldsymbol{\beta}_c^{(t)} - X_s \boldsymbol{\beta}_s^{(t)})$
    $\boldsymbol{\alpha}_2^{(t)} = \kappa_2^{(t-1)} H^{(t-1)-} (\boldsymbol{y}^{\star(t-1)} - X_c \boldsymbol{\beta}_c^{(t)} - X_s \boldsymbol{\beta}_s^{(t)})$
    $\kappa_1^{(t)}, \kappa_2^{(t)} = argmax \log L(\kappa_1, \kappa_2 | \boldsymbol{y}^{\star(t-1)})$
    Update $\boldsymbol{\mu}^{(t)}$, $V^{(t)}$, $H^{(t)}$ and $\boldsymbol{y}^{\star(t)}$
  end while
end procedure

And then, to update the estimates of κ_1 and κ_2, we maximize the profile pseudo-likelihood

$$\begin{aligned}\log L(\kappa_1, \kappa_2 | \boldsymbol{y}^\star) \quad \propto \quad & -\frac{1}{2} \log \left| \kappa_1 \Sigma + \kappa_2 I + \widehat{V}^{-1} \right| \\ & -\frac{1}{2} (\boldsymbol{y}^\star - X_c \widehat{\boldsymbol{\beta}}_c - X_s \widehat{\boldsymbol{\beta}}_s)^T \left(\kappa_1 \Sigma + \kappa_2 I + \widehat{V}^{-1} \right)^{-1} (\boldsymbol{y}^\star - X_c \widehat{\boldsymbol{\beta}}_c - X_s \widehat{\boldsymbol{\beta}}_s),\end{aligned}$$

obtaining the estimates $\widehat{\kappa}_1, \widehat{\kappa}_2 = argmax \log L(\kappa_1, \kappa_2 | \boldsymbol{y}^\star)$.

The pseudo-likelihood algorithm proceeds iteratively updating the parameters until convergence. Algorithm 1 summarizes the pseudo-likelihood approach for baseline models.

S1.2. Model fitting for non-baseline models

In each BG2 step, a baseline model is fitted with the pseudo-likelihood approach presented in Section 1.1. This results in estimated variance parameters and a vector of adjusted observations $\boldsymbol{y}^\star$. After that, these variance parameter estimates and vector of adjusted observations $\boldsymbol{y}^\star$ are used to fit the non-baseline models in that BG2 step. As a result, fitting a non-baseline model does not have any iteration, but just computes the estimate of the regression coefficients with the formula $\widehat{\boldsymbol{\beta}} = (X^T \widehat{H}^{-1} X)^{-1} X^T \widehat{H}^{-1} \boldsymbol{y}^\star$. The matrix H, which is a function of the variance parameters, is estimated with the baseline model at the beginning of the respective BG2 step, and then it remains fixed for all non-baseline models. Hence, the eigen decomposition of H can be computed at the beginning of the BG2 step and the same linear algebra trick used in EMMAX can be used in BG2. Therefore, fitting non-baseline models is super fast.

The baseline model differs in the screening step and in the model selection step. In the screening step, the baseline model is the null model with no SNPs, that is, X_s is not included in the null model. In the model selection step, the baseline model is the full model with X_s containing all candidate SNPs identified in the screening step.

S2. Results of simulation study of count data simulated with human genome

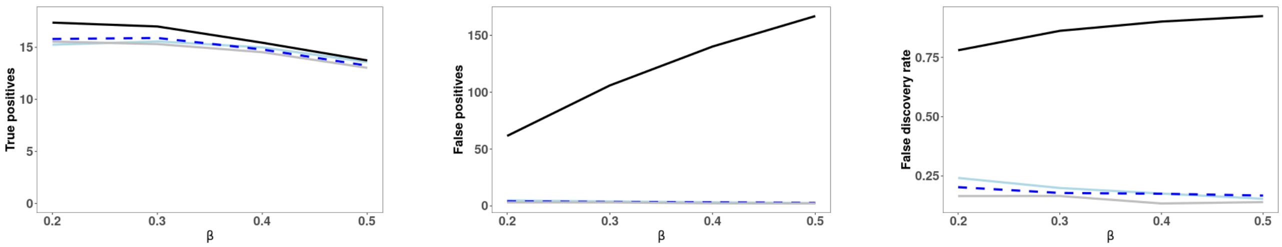

(a) True positives (b) False positives (c) False discovery rate

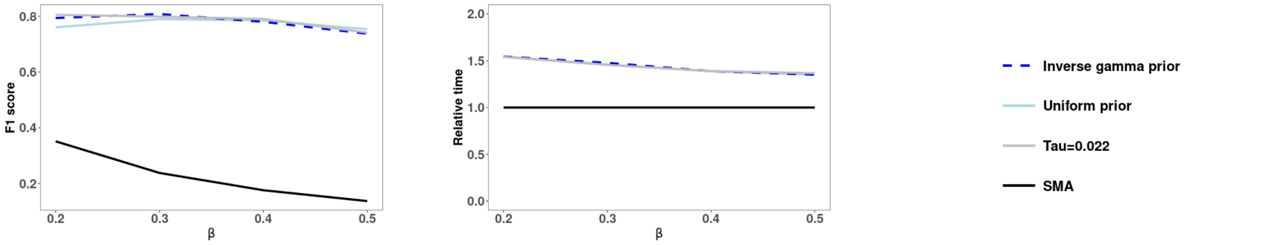

(d) F1 score (e) Relative time

Figure S1: Results for simulated count data based on human genome. SNP search performance of four methods (SMA and three proposed BG^2 methods: uniform prior for τ, inverse gamma prior for τ, and fixed $\tau = 0.022$) averaged over 100 datasets under each parameter setting $\beta = 0.2, 0.3, 0.4, 0.5$ respectively. Intercept $\beta_0 = -0.5$, variance component for kinship random effects $\kappa_1 = 0.1$, variance component for overdispersion random effects $\kappa_2 = 0.05$. Five criteria: True positives (TP), false positives (FP), false discovery rate (FDR), F1 score (F1) and Relative time with respect to SMA.

S3. Boxplots of TP, FP, FDR, and F1 in the simulation studies

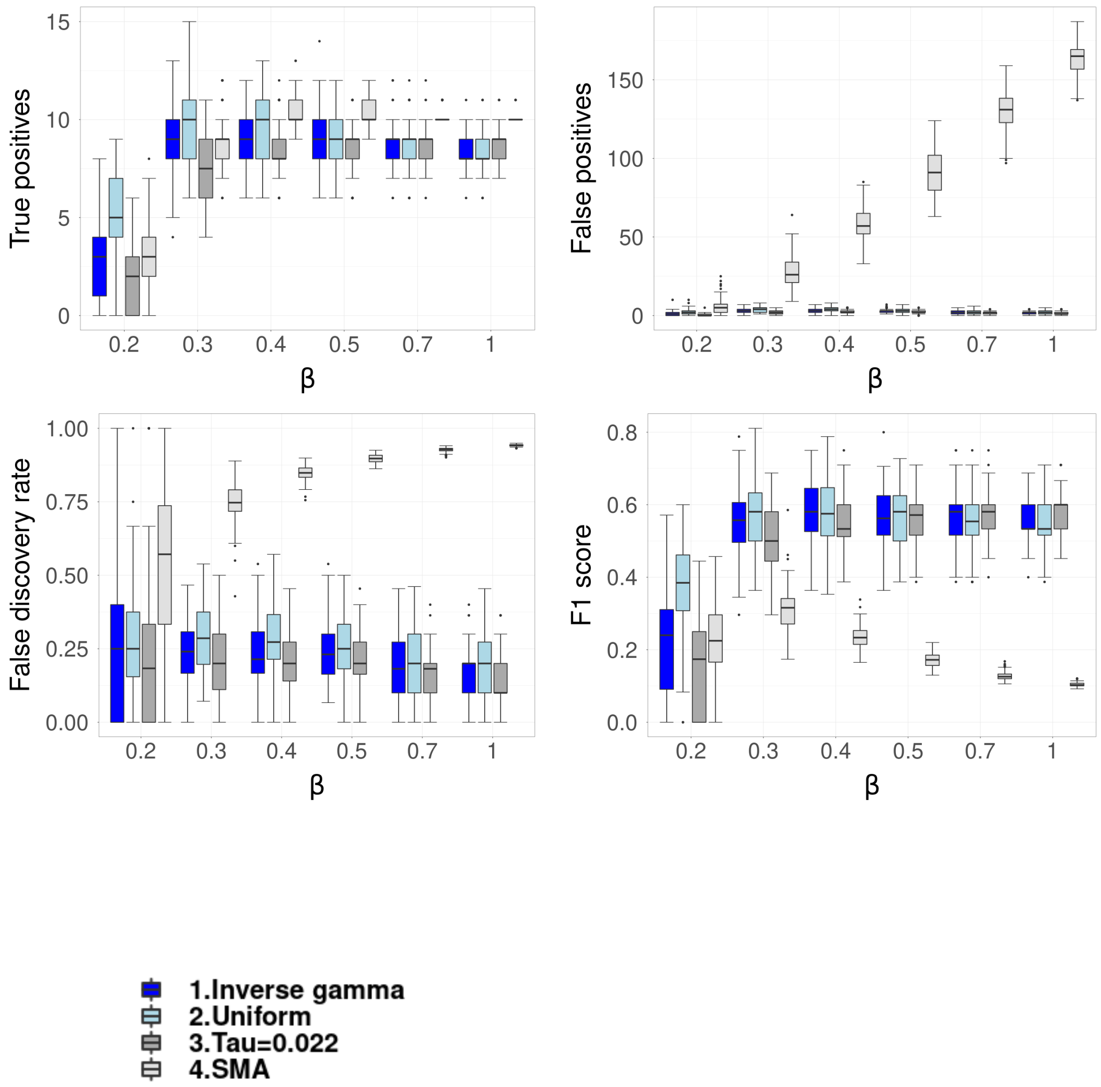

Figure S2: Results for simulated binary data based on human genome. SNP search performance of four methods (SMA and three proposed BG2 methods: uniform prior for τ, inverse gamma prior for τ, and fixed $\tau = 0.022$) for 100 simulated datasets under each parameter setting $\beta = 0.2, 0.3, 0.4, 0.5, 0.7, 1$ respectively. Boxplots of four criteria: True positives (TP), false positives (FP), false discovery rate (FDR), and F1 score (F1).

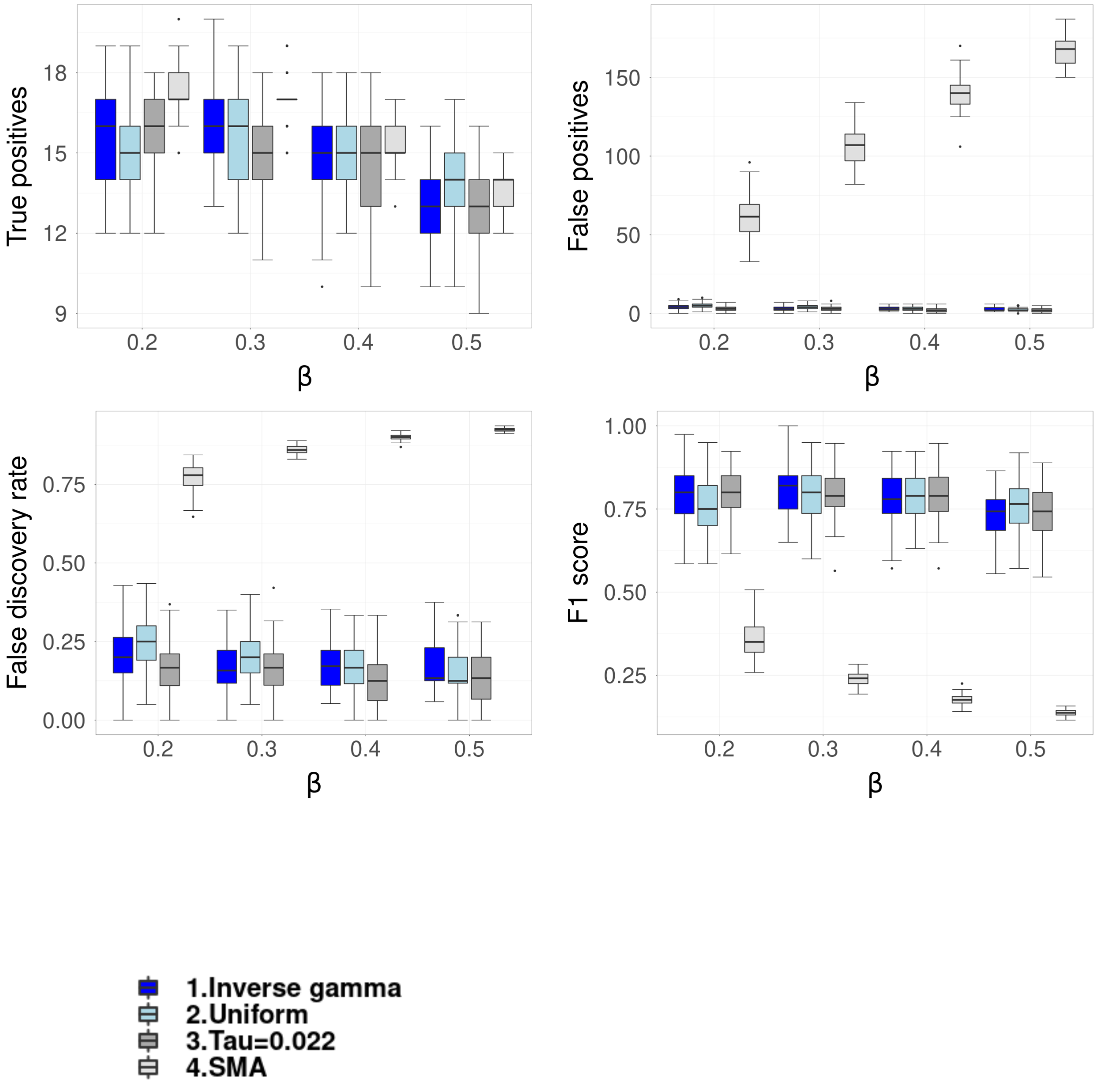

Figure S3: Results for simulated count data based on human genome. SNP search performance of four methods (SMA and three proposed BG2 methods: uniform prior for τ, inverse gamma prior for τ, and fixed $\tau = 0.022$) for 100 simulated datasets under each parameter setting $\beta = 0.2, 0.3, 0.4, 0.5$ respectively. Boxplots of four criteria: True positives (TP), false positives (FP), false discovery rate (FDR), and F1 score (F1).

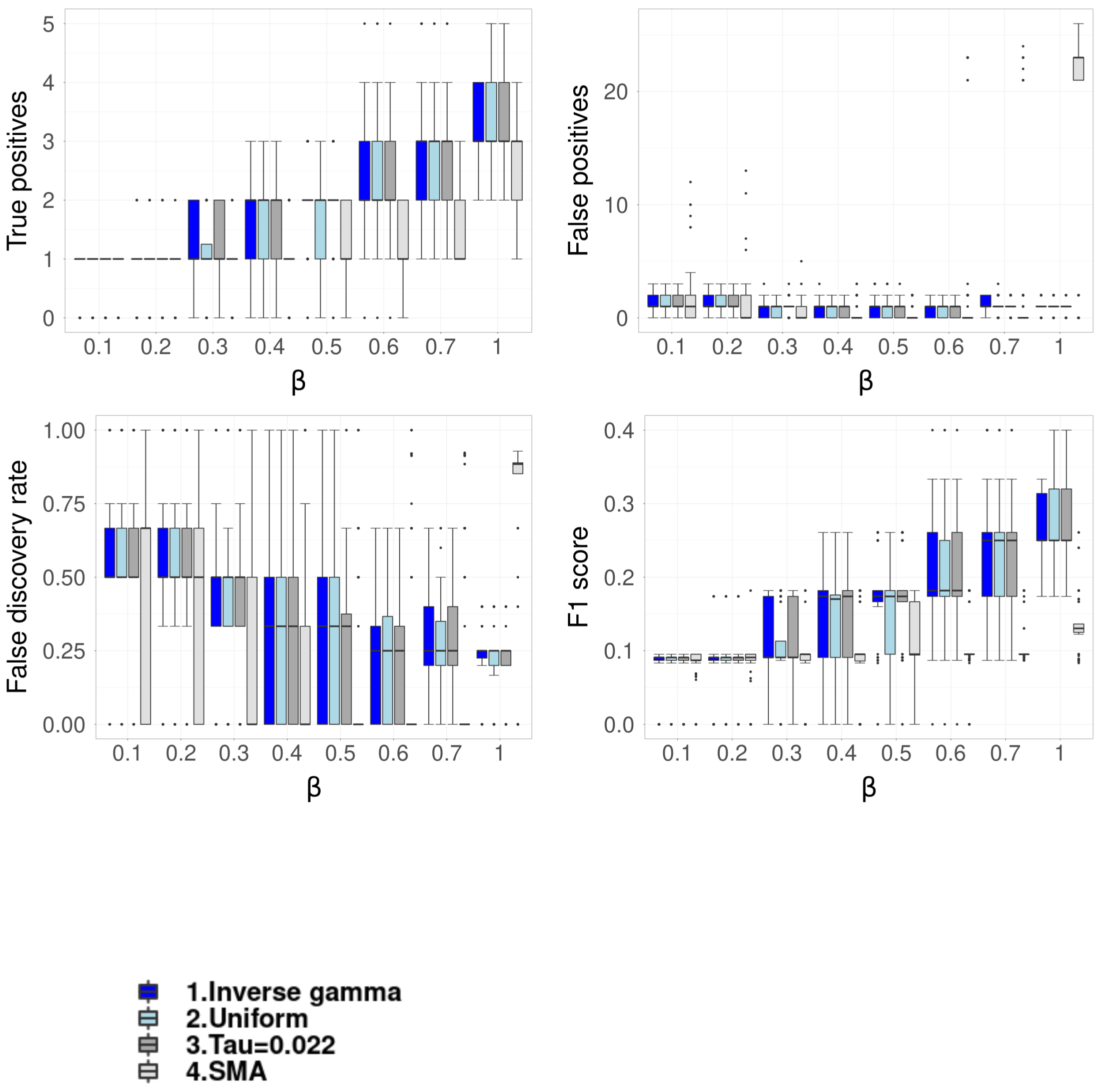

Figure S4: Results for simulated count data based on A. Thaliana genome. SNP search performance of four methods (SMA and three proposed BG2 methods: uniform prior for τ, inverse gamma prior for τ, and fixed $\tau = 0.022$) for 100 simulated datasets under each parameter setting $\beta = 0.1, 0.2, 0.3, 0.4, 0.5, 0.6, 0.7, 1$ respectively. Boxplots of four criteria: True positives (TP), false positives (FP), false discovery rate (FDR), and F1 score (F1).

S4. Robustness of BG2 when dealing with imbalanced binary data or highly skewed count data

In the original simulation studies for count data presented in Section 4.2, we have highly skewed count data. To visualize that, Figure S5(a) shows that when $\beta = 0.1$ the count data are skewed. In addition, Figure S5(b) shows that when $\beta = 0.7$ the count data are tremendously skewed. Table S1 shows that skewed count data do not affect variable selection. As a matter of fact, when BG2 is applied to more skewed data ($\beta = 0.7$) the performance of BG2 improves with higher TP, lower FP and FDR, and higher F1 score.

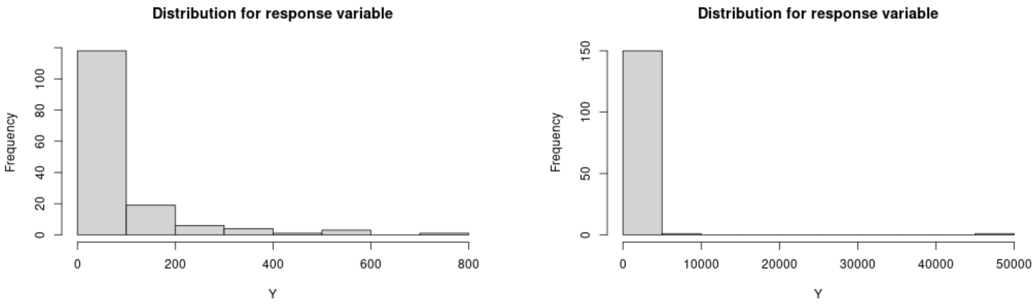

(a) $\beta = 0.1$ (b) $\beta = 0.7$

Figure S5: Histograms for two simulated count dataset from Section 4.2. The simulation setting has 10 Causal SNPs, 5 SNPs with coefficient 0.2, 5 SNPs with coefficient β. (a) $\beta = 0.1$. (b) $\beta = 0.7$.

	Method	TP	FP	FDR	F1
$\beta = 0.1$	Inverse gamma	0.82	1.37	0.63	0.13
	Uniform	0.82	1.36	0.62	0.13
	$\tau = 0.022$	0.83	1.36	0.62	0.14
	SMA	0.88	1.53	0.63	0.14
$\beta = 0.7$	Inverse gamma	2.90	1.11	0.28	0.41
	Uniform	2.70	1.03	0.28	0.39
	$\tau = 0.022$	2.78	1.02	0.27	0.40
	SMA	1.28	2.28	0.64	0.19

Table S1: Performance of BG2 and SMA when data are skewed or tremendously skewed. When $\beta = 0.1$, count data are skewed. When $\beta = 0.7$, count data are tremendously skewed.

The binary data we have in the original simulation study presented in Section 4.1 is almost balanced, with about 56% 0s and 44% 1s. We have added a new simulation study with $\beta_0 = 2$, which is highly imbalanced with about 29% 0s and 71% 1s. Table S2 shows that BG2 is robust to imbalanced data, and even performs slightly better when the data are imbalanced.

	Method	TP	FP	FDR	F1
$\beta_0 = -0.5$	Inverse gamma	8.50	1.68	0.17	0.84
	Uniform	8.36	1.96	0.19	0.82
	$\tau = 0.022$	8.60	1.51	0.15	0.86
	SMA	10.04	164.25	0.94	0.11
$\beta_0 = 2$	Inverse gamma	8.77	1.50	0.15	0.87
	Uniform	8.74	1.58	0.15	0.86
	$\tau = 0.022$	8.73	1.34	0.13	0.87
	SMA	10.01	156.53	0.94	0.11

Table S2: Performance of BG2 and SMA when data are balanced or imbalanced. When $\beta_0 = -0.5$, binary data are balanced. When $\beta_0 = 2$, binary data are imbalanced.

S5. Robustness of BG2 to genome spacing of SNPs

To verify the robustness of BG2 to genome spacing of SNPs, we have added three new simulation studies that we name SIM1, SIM2, and SIM3. These simulation studies expand the simulation study from Section 4.1 for binary data based on human genome.

In the first simulation study SIM1, we generate data from 20 causal SNPs, which are from 4 clusters. In each cluster, there are 5 causal SNPs. Each cluster has a length of 30000 bp. In two clusters, two SNPs have large coefficient $\beta = 1$. In another two clusters, three SNPs have large coefficient $\beta = 1$. All the other SNPs have small coefficient 0.2 or -0.2. For reference, simulation study SIM0 in the Table S3 is the simulation study in Section 4.1, which has the same parameter setting except that 20 SNPs are evenly spaced.

In the second simulation study SIM2, we generate data from 10 causal SNPs, which are from 2 clusters. In each cluster, there are 5 causal SNPs. Each cluster has a length of 30000 bp. In one cluster, two SNPs have large coefficient $\beta = 1$. In another two clusters, three SNPs have large coefficient $\beta = 1$. All the other SNPs have small coefficient 0.2 or -0.2.

In the third simulation study SIM3, we generate data from 5 causal SNPs, which are from only one cluster. The length of the cluster is 30000 bp. Three SNPs have large coefficient $\beta = 1$. One SNP has coefficient 0.2, and another SNP has coefficient -0.2.

Table S3 shows that BG2 can detect almost all SNPs with large coefficient. The number of causal SNPs and the position of SNPs do not alter the performance of the method BG2. Comparing SIM0 and SIM1, BG2 for clustering causal SNPs has lower FP and FDR, and higher F1. Comparing SIM1, SIM2, and SIM3, small number of causal SNPs make BG2 have lower FDR and higher F1 score.

Simulation	Method	TP	FP	FDR	F1
SIM0	Inverse gamma	8.50	1.68	0.17	0.56
	Uniform	8.36	1.96	0.19	0.55
	$\tau = 0.022$	8.60	1.51	0.15	0.57
	SMA	10.04	164.25	0.94	0.10
SIM1	Inverse gamma	8.68	0.36	0.04	0.60
	Uniform	8.66	0.44	0.05	0.60
	$\tau = 0.022$	8.56	0.34	0.04	0.59
	SMA	17.11	75.00	0.81	0.31
SIM2	Inverse gamma	4.94	0.02	0.004	0.66
	Uniform	4.92	0.02	0.006	0.66
	$\tau = 0.022$	4.91	0.03	0.004	0.66
	SMA	8.53	47.22	0.847	0.26
SIM3	Inverse gamma	2.88	0.01	0.003	0.73
	Uniform	2.92	0.01	0.003	0.74
	$\tau = 0.022$	2.79	0.00	0.000	0.72
	SMA	5.00	48.50	0.907	0.17

Table S3: Robustness to genome spacing of SNPs. Performance of BG2 and SMA when the number of causal SNPs decreases and the SNPs are clustered. SIM0: 20 evenly spaced causal SNPs. SIM1: 20 causal SNPs in four clusters. SIM2: 10 causal SNPs in two clusters. SIM3: 5 causal SNPs in one cluster.

S6. Sensitivity of BG2 to parameter values

To study the sensitivity of BG2 to the values of parameters, we have added three new simulation studies.

The first of these simulation studies is SIM4 where, instead of 0.2 or -0.2, the regression coefficients for 10 causal SNPs are 0.4 and -0.4. Other 10 causal SNPs' coefficient β have six parameter settings: 0.2, 0.3, 0.4, 0.5, 0.7 and 1. Intercept $\beta_0 = -0.5$. Variance component for kinship random effects $\kappa = 0.15$. Figure S6 presents results for the SIM4 simulation study. Compared with Figure 1, Figure S6 shows that when all 20 causal SNPs' coefficient are equal to 0.4, BG2 can detect about 16 causal SNPs. Otherwise, BG2 can detect about 10 SNPs with relative large coefficient. In addition, BG2 has higher F1 score in Figure S6 than in Figure 1.

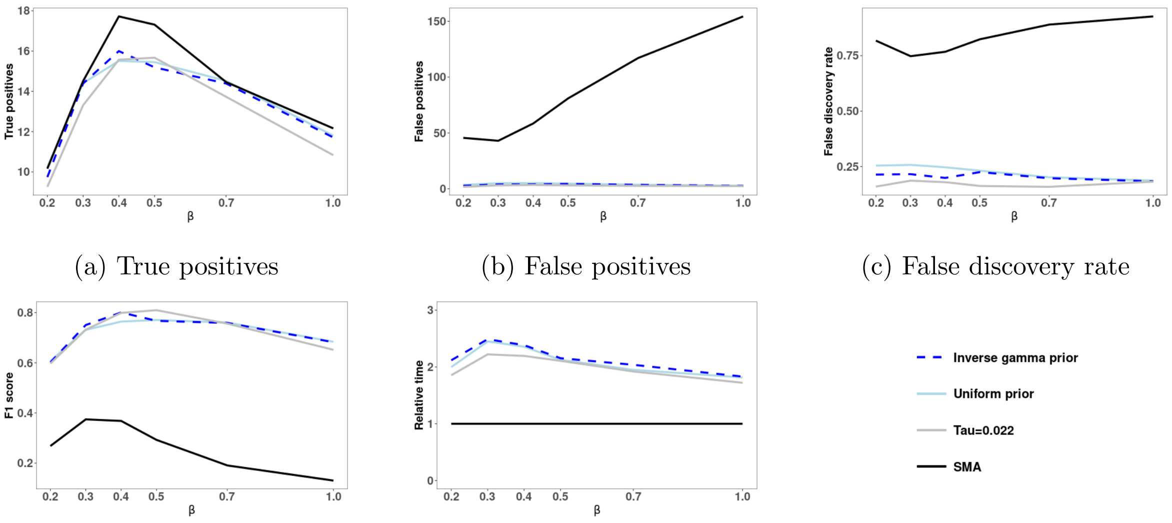

(a) True positives (b) False positives (c) False discovery rate

(d) F1 score (e) Relative time

Figure S6: Results of simulation study SIM4. Performance of BG2 and SMA. Regression coefficients for 10 causal SNPs are 0.4 and -0.4. Another 10 causal SNPs' coefficient β have six parameter settings: 0.2, 0.3, 0.4, 0.5, 0.7 and 1. Intercept $\beta_0 = -0.5$. Variance component for kinship random effects $\kappa = 0.15$.

The second of these simulation studies is SIM5 where, instead of $\beta_0 = -0.5$, the intercept is $\beta_0 = 1$. We have 20 causal SNPs. The regression coefficients for 10 causal SNPs are 0.2 and -0.2. Another 10 causal SNPs' coefficient β have six parameter settings: 0.2, 0.3, 0.4, 0.5, 0.7 and 1. Variance component for kinship random effects $\kappa = 0.15$. Figure S7 presents the results for SIM5. The results presented in Figure S7 look similar to those presented in Figure 1. Thus, BG2 does not seem to be sensitive to changes in β_0.

The third of these simulation studies is SIM6 where, instead of $\kappa = 0.15$, the variance component of the kinship random effects is $\kappa = 0.3$. We have 20 causal SNPs. The regression coefficients for 10 causal SNPs are 0.2 and -0.2. Another 10 causal SNPs' coefficient β have six parameter settings: 0.2, 0.3, 0.4, 0.5, 0.7 and 1. Intercept $\beta_0 = -0.5$. Figure S8 presents the results for SIM6. The

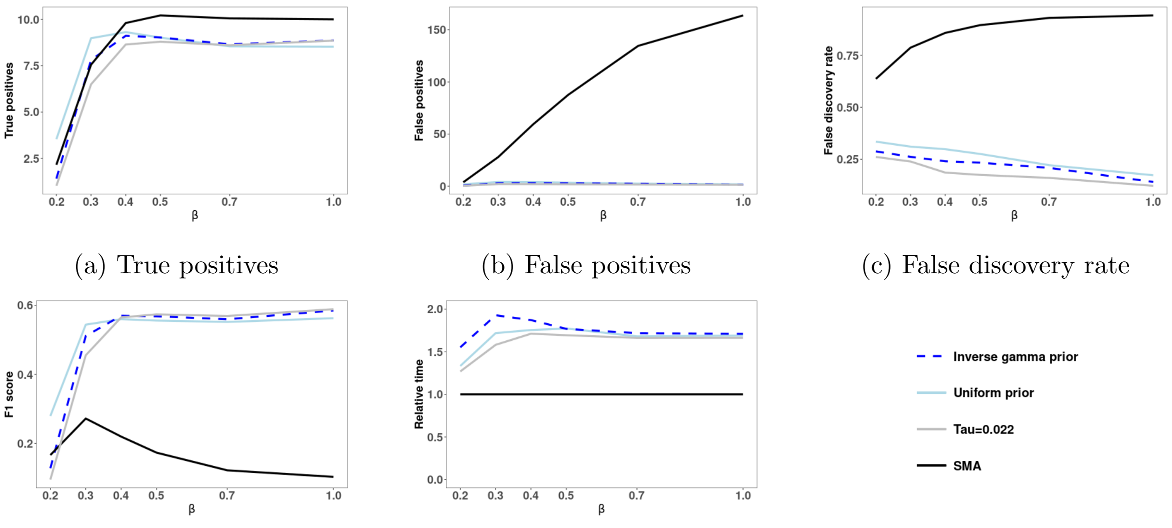

(a) True positives (b) False positives (c) False discovery rate

(d) F1 score (e) Relative time

Figure S7: Results of simulation study SIM5. Performance of BG2 and SMA. Intercept $\beta_0 = 1$. Generate data from 20 causal SNPs. The regression coefficients for 10 causal SNPs are 0.2 and -0.2. Another 10 causal SNPs' coefficient β have six parameter settings: 0.2, 0.3, 0.4, 0.5, 0.7 and 1. Variance component for kinship random effects $\kappa = 0.15$.

results in Figure S8 seem to be fairly similar to those in Figure 1. Thus, BG2 performs similarly when κ changes from 0.15 to 0.3.

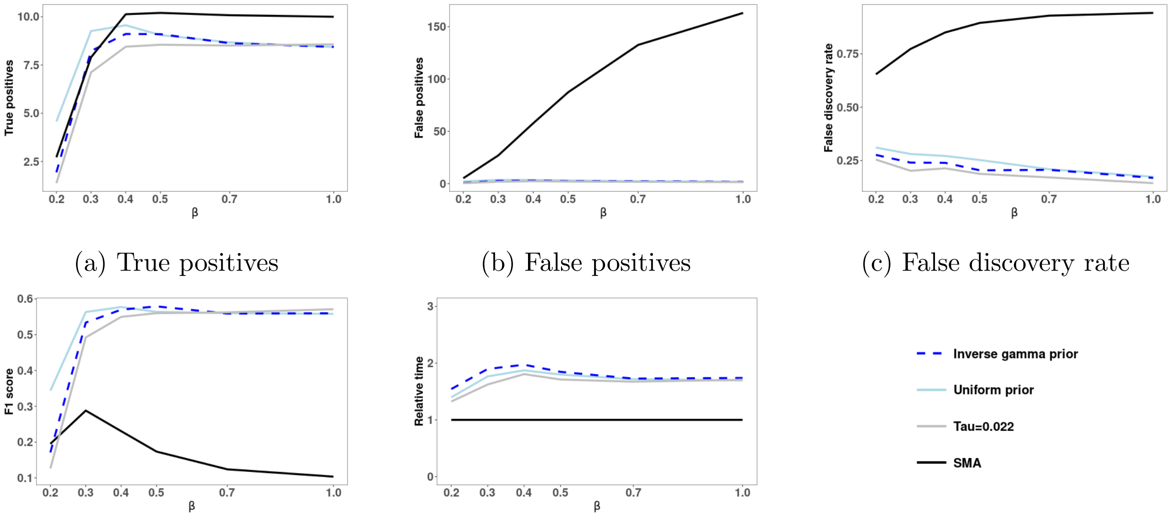

(a) True positives (b) False positives (c) False discovery rate

(d) F1 score (e) Relative time

Figure S8: Results of simulation study SIM6. Performance of BG2 and SMA. Variance component for kinship random effects $\kappa = 0.3$. Generate data from 20 causal SNPs. The regression coefficients for 10 causal SNPs are 0.2 and -0.2. Another 10 causal SNPs' coefficient β have six parameter settings: 0.2, 0.3, 0.4, 0.5, 0.7 and 1. Intercept $\beta_0 = -0.5$.

S7. Calibration of the pseudo-likelihood approach

The BG2 approach does not provide p-values. To check if the pseudo-likelihood approach is calibrated, we compute p-values based on the pseudo-likelihood approach for two datasets from Sections 4.1 and 4.2 that do not have any causal SNP. In this case, if the pseudo-likelihood approach is calibrated then the distribution of the p-values should be a uniform distribution. Figure S9 presents a Q-Q plot of p-values from one binary dataset in Section 4.1 whereas Figure S10 presents a Q-Q plot of p-values from one count dataset in Section 4.2. It is clear from the figures that in both cases the p-values have a uniform distribution. Therefore, the pseudo-likelihood approach is calibrated.

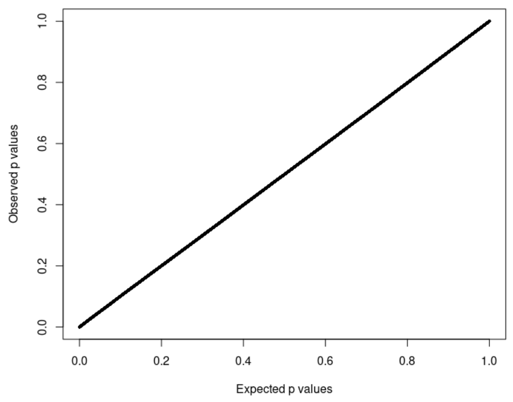

Figure S9: Calibration of the pseudo-likelihood approach. Binary data simulated from human genome data. Q-Q plot of p-values based the pseudo-likelihood approach.

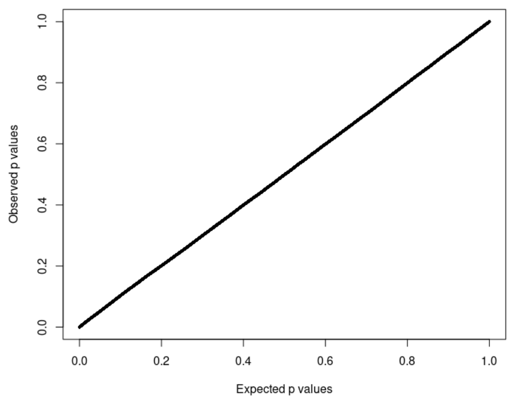

Figure S10: Calibration of the pseudo-likelihood approach. Count data simulated from A. Thaliana genome data. Q-Q plot of p-values based the pseudo-likelihood approach.

S8. Histograms of the response variables in the case studies

Figures S11, S12, and S13 present the histograms of the response variables for each of the three case studies. From these figures, it is clear that the count response variables in the case studies presented in Sections 5.1 and 5.3 are skewed. However, the new Section S4 from the Supplementary Material shows that BG2 can deal with imbalanced data without difficulties, and that the performance of BG2 improves as the level of skewness increases. In addition, the binary response variable from the case study presented in Section 5.2 is imbalanced. However, the new Section S4 shows that BG2 is robust to imbalanced data, and even performs slightly better when the data are imbalanced.

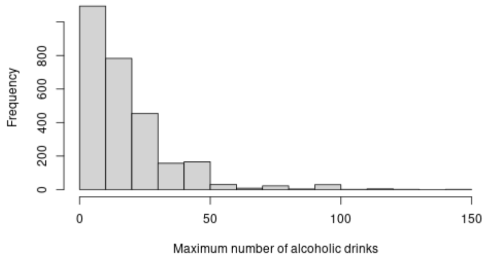

Figure S11: Case study from Section 5.1: Maximum number of alcoholic drinks. Histogram of the maximum number of alcoholic drinks.

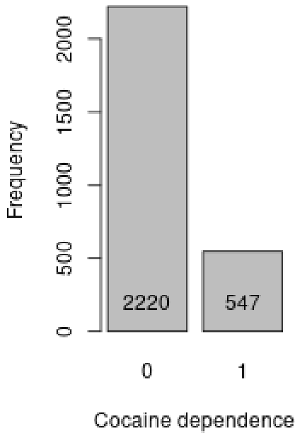

Figure S12: Case study from Section 5.2: Cocaine dependence. Histogram of the response variable cocaine dependence that is equal to 1 if the subject is cocaine dependent and 0 otherwise.

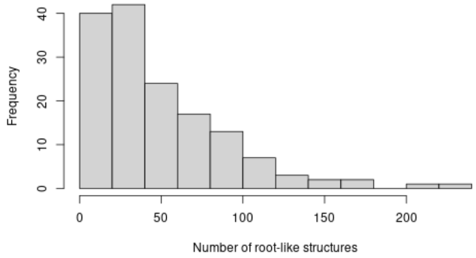

Figure S13: Case study from Section 5.3: Root-like structures in *A. Thaliana*. Histogram of number of root-like structures in A. Thaliana.
